# Histology-specific standardized incidence ratio improves the estimation of second primary lung cancer risk

**DOI:** 10.1186/s12916-024-03398-9

**Published:** 2024-05-03

**Authors:** Marian Eberl, Luana F. Tanaka, Klaus Kraywinkel, Stefanie J. Klug

**Affiliations:** 1 TUM School of Medicine and Health, Technical University of Munich, Georg-Brauchle-Ring 56, Munich, 80992 Germany; 2 German Centre for Cancer Registry Data, Robert Koch-Institut, Nordufer 20, Berlin, 13353 Germany

**Keywords:** Second primary cancer, Lung cancer, Cancer epidemiology, Cancer registry data, Standardized incidence ratio

## Abstract

**Background:**

Lung cancer (LC) survivors are at increased risk for developing a second primary cancer (SPC) compared to the general population. While this risk is particularly high for smoking-related SPCs, the published standardized incidence ratio (SIR) for lung cancer after lung cancer is unexpectedly low in countries that follow international multiple primary (IARC/IACR MP) rules when compared to the USA, where distinct rules are employed. IARC/IACR rules rely on histology-dependent documentation of SPC with the same location as the first cancer and only classify an SPC when tumors present different histology. Thus, SIR might be underestimated in cancer registries using these rules. This study aims to assess whether using histology-specific reference rates for calculating SIR improves risk estimates for second primary lung cancer (SPLC) in LC survivors.

**Methods:**

We (i) use the distribution of histologic subtypes of LC in population-based cancer registry data of 11 regional cancer registries from Germany to present evidence that the conventional SIR metric underestimates the actual risk for SPLC in LC survivors in registries that use IARC/IACR MP rules, (ii) present updated risk estimates for SPLC in Germany using a novel method to calculate histological subtype-specific SIRs, and (iii) validate this new method using US SEER (Surveillance, Epidemiology, and End Results Program) data, where different MP rules are applied.

**Results:**

The adjusted relative risk for lung cancer survivors in Germany to develop an SPLC was 2.98 (95% CI 2.53–3.49) for females and 1.15 (95% CI 1.03–1.27) for males using the novel histology-specific SIR. When using IARC/IACR MP rules, the conventional SIR underestimates the actual risk for SPLC in LC survivors by approximately 30% for both sexes.

**Conclusions:**

Our proposed histology-specific method makes the SIR metric more robust against MP rules and, thus, more suitable for cross-country comparisons.

**Supplementary Information:**

The online version contains supplementary material available at 10.1186/s12916-024-03398-9.

## Background

The International Agency for Research on Cancer (IARC) collects, harmonizes, and publishes data on cancer incidence worldwide. According to recent estimates, 2.2 million lung cancer (LC) cases were newly diagnosed worldwide in 2020, comprising 11.4% of all cancer diagnoses, with an increasing incidence trend among women [1, 2]. Lung cancer survivors are at an overall increased risk for developing a second primary cancer (SPC) compared to the general population. Subsequent tumors of the oral cavity, pharynx, digestive, and respiratory organs are of particular importance because a large proportion of survivors are long-time smokers who continue smoking [3–6]. However, for some countries, such as Germany or the UK, published standardized incidence ratios (SIR) for second primary lung cancer (SPLC) after lung cancer are unexpectedly low and even suggest a risk reduction for diseased males compared to the general population (e.g., 0.83 and 2.06 for males and females in Germany [7]; 1.47 in the UK (not stratified by sex) [8]). Data from the USA, in contrast, indicate that lung cancer survivors are three to five times more likely to have another lung tumor than the general population, which has lower smoking rates (SIR = 3.38 for males and 4.85 for females) [9].

An essential difference between the US data with a high risk for SPLC and German data with a much lower risk is that national registries apply different rules defining SPLCs. The German registries, and most other international registries, follow the rules for multiple primary cancers (MP rules) defined by the International Agency for Research on Cancer and the International Association for Cancer Registration (IARC/IACR) [10]. They define that only 1 primary cancer per organ can be diagnosed in a patient’s lifetime unless multiple tumors are “histologically different.” IARC/IACR MP rules define 8 different histological groups for LC [11]. In contrast, North American cancer registries follow the rules of the Surveillance, Epidemiology, and End Results Program (SEER) [12], considering timing, laterality, and more detailed histology with 34 different groups. Consequently, a second lung adenocarcinoma in a person fully recovered from a previous lung adenocarcinoma diagnosis would not be classified as an SPLC according to IARC/IACR rules, even if the 2 events are 10 years apart. In contrast, SEER would classify the diagnosis as an SPLC because any tumor is considered a new primary LC after three disease-free years. Additionally, even if the disease-free period since the initial diagnosis is shorter, an SPLC would be registered in the US registries if the second adenocarcinoma had an ICD-O-3 histology code that was different at the third number (e.g., bronchiolo-alveolar adenocarcinoma 8250 and papillary adenocarcinoma 8260). Additional file 1: Table S1 shows a detailed comparison of IARC/IACR and SEER MP rules.

Estimating risk for same-site SPC has been an unsolved methodological issue for many years, and experts have warned that using SIR can be misleading [13]. Cross-country comparison is difficult due to varying registration practices [14]. Consequently, recent high-quality publications have not presented respective estimates [4, 15] or highlighted that low-risk estimates contradict increased exposure and may be an artifact of calculating SIR as a relative risk estimate [7]. For cancer registries using IARC/IACR MP rules, SIRs might be underestimated due to the histology-dependent documentation of SPC with the same location as the first cancer. We want to narrow this methodological gap in cancer epidemiology by proposing an adapted risk estimate for same-site SPC that considers the histologic subtype of the first cancer.

### Objective

This study aims to assess whether using histology-specific reference rates for estimating the expected number of cases improves the estimation of SIR for SPLC in LC survivors. Therefore, this analysis (i) demonstrates how the conventional SIR metric underestimates the risk for SPLC in LC survivors documented in cancer registries that use IARC/IACR MP rules and (ii) proposes a novel method to calculate histology-specific SIRs to reduce bias and presents updated risk estimates for SPLC in Germany. Finally, we (iii) validate our results by comparing how this new method performs when estimating risk for SPLC in the USA, where multiple primaries are registered following SEER rules.

## Methods

### Data

This study uses pooled data from 11 regional German population-based cancer registries (PBCRs) that follow IARC/IACR MP rules, covering ~ 50% (40 million) of the German population [16]. The analysis dataset included all LC survivors diagnosed between 2002 and 2013 who survived at least 6 months after the initial diagnosis. The data source has been previously described [7, 17]; details on case selection and data modifications are described in Additional file 1: Table S2 and Table S3.

As a validation dataset, we pooled data from 17 SEER regions that started registration before 2002 [18]. Details on the included regions and data quality can be found in Additional file 1: Table S4. Methodological aspects of the SEER database were described by Curtis et al. [19].

We set the end of follow-up in both data sources to 31 December 2014 to ensure comparability. We counted SPLC (ICD-10 code C34) and excluded unusual morphology codes for LC that were likely miscoded metastases (codes 8263, 8290, 8720, 8815, 8933, 9050, and 9133).

Our definition of histologically different groups is based on the International Classification of Diseases for Oncology, third edition (ICD-O-3), revision 1, published in 2013 [11]. The four-digit morphology codes were converted into histology groups as listed in Additional file 1: Table S5. To present aggregated results, we additionally grouped the histologic subtypes of LC into squamous cell carcinoma (SCC), adenocarcinoma (AC), small-cell carcinoma (SCLC), large-cell carcinoma (LCC), and other and unspecified (O&U) as suggested by the WHO classification of thoracic tumors [20].

### Statistical analysis

#### Estimating the size of bias for using standard SIR

We simulated various scenarios to estimate the size of bias introduced by using general population reference rates for calculating SIR (ratio of observed to expected cancers O/E) of same-site SPC when IARC/IACR MP rules are applied. This bias occurs because the count for *O* excludes same-histology SPLC, while the reference rates used to calculate *E* include same-histology. Our first simulation setting is that the baseline risk of LC survivors to develop an SPLC is the same as that for the general population (${{\text{SIR}}}_{{\text{real}}}=1.0$). We determined the proportions of histologically different LC groups ${p}_{{{\text{hist}}}_{j}}$ in the analysis dataset for all index LC cases stratified by sex and assumed that the SPLC would have the same histology group distribution as for the first cancer. To obtain the simulated ${{\text{SIR}}}_{{\text{simIARC}}}$, we multiplied each stratum of obtained expected numbers with a sex- and histology-group correction factor ${x}_{{{\text{hist}}}_{j}}=1-{p}_{{{\text{hist}}}_{j}}$ for combinations of LC and SPLC that are not possible in our observed cases. In addition to the scenario of no risk difference, we also simulate the impact of this bias when SPLC risk for LC survivors is double that of the general population (${{\text{SIR}}}_{{\text{real}}}=2.0$) and a risk increase equivalent to data published by Thakur et al. for US male (${{\text{SIR}}}_{{\text{real}}}=3.38$) and female (${{\text{SIR}}}_{{\text{real}}}=4.85$) LC survivors [9]. Further details on the simulations can be found in Additional file 1: Section S6.

#### Calculating a histology-specific SIR

To calculate the conventional SIR (ratio of observed to expected SPLC cases), referred to as SIR1_raw_, we used age-, sex-, region-, and period-specific general reference rates obtained from the full sample of German PBCR data, including cases with a diagnosis based on a death certificate only (DCO).

**Fig. 1:**
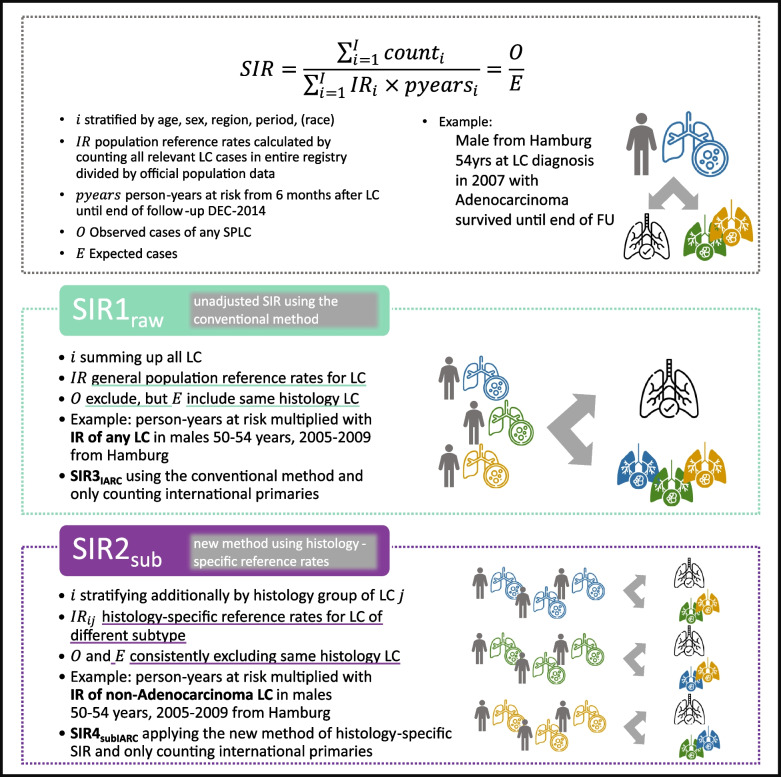
Comparison of conventional method to calculate standardized incidence ratio (SIR1_raw_) and newly proposed method to stratify by histology group of LC and use group-specific reference rates excluding same histology (SIR2_sub_). The top gray dotted box shows the general formula for SIR, explains the operationalization for each variable in our study setting, and presents an example assessing the risk of SPLC for a survivor with prior adenocarcinoma LC. Icons are visualizing this example. The middle green dotted box explains that in the conventional SIR method, the sample patient is assessed together (unstratified) with the overall risk of other primary LC histologies, and the risk is assessed including all SPLC histologies in the reference rates. In contrast, the bottom violet dotted box shows that the newly proposed method additionally stratifies risk calculation by LC histology, and only different histology SPLCs are counted when calculating the reference rates. *Notes*: Gray arrows mark the option to either not get an SPLC (healthy lung depicted uncolored with a check mark) or be diagnosed with an SPLC of different histology (i.e., different color). Blue, green, and yellow symbolize different histology groups (for simplicity, we only show three). Colored unfilled lungs depict the histology group of the first LC; colored filled lungs depict the histology group of SPLC

Next, we propose a novel method to stratify SIR calculations by histology group of LC (e.g., adenocarcinomas) and use group-specific reference rates excluding same-histology SPLC. The concepts of the conventional SIR1_raw_ and the newly proposed histological subtype-specific SIR2_sub_ are explained in Fig. 1. Most importantly, calculating histological subtype-specific SIR removes the discrepancy in the conventional method of excluding same-histology group tumors from the observed count (O)—due to IARC/IACR MP rules—while including those same-histology tumors in the reference rates, i.e., expected cases. When calculating the SIR2_sub_, we always stratified by the histology group of the first tumor. Then, we used histology-specific reference rates ${{\text{IR}}}_{ij}$ that excluded the same histology group from the incidence. In settings where same-histology SPCs occur in the data (i.e., PBCR not applying IARC/IACR MP rules), those cancers are not counted as observed.

Finally, for all SIR, we aggregated $O$ and $E$ for the total available follow-up from 6 months to a maximum of 13 years and always reported sex-specific estimates. We calculated 95% confidence intervals (CIs) as described by Breslow and Day [21].

#### Validation analysis using SEER data

To validate our newly proposed method, we apply the SIR2_sub_ measure to cancer registry data that does not follow IARC/IACR MP rules and assess whether this introduces unintended bias. We present descriptive statistics on the validation dataset and interpret comparability with the analysis dataset. Then, we calculate the unadjusted SIR1_raw_ for the USA and SIR2_sub_ using the same method described above. Furthermore, we obtained individual-level information on which cancer cases in the SEER data fulfill the IARC/IACR MP rules (variable INTPRIM). We recalculated the unadjusted SIR for SPLC counted under IARC/IACR rules (SIR3_IARC_) and histology-specific SIR (SIR4_subIARC_) using patient-level data to receive updated reference rates. To assess the validity of our histology-specific SIR method, we compare the following:

- SIR2_sub_ and SIR4_subIARC_ in the validation dataset with the expectation that they are very similar and lie between SIR1_raw_ and SIR3_IARC_ (this would show that the new method delivers a robust estimate, independent of MP rules applied)
- SIR2_sub_ estimates between Germany and the USA with the expectation that they should be closer than SIR1_raw_ estimates (this is relevant for a scenario where a registry’s abidance by IARC/IACR rules is unknown and we want to receive an estimate suitable for cross-country comparison)
- SIR2_sub_ estimates from Germany to SIR4_subIARC_ estimates from the USA to determine the size of residual cross-country differences

#### Sensitivity analyses

For sensitivity analyses, we restricted our dataset to six German PBCRs with a low DCO rate (smaller than 10%)—an indicator for higher registration quality—and assessed whether this influences the overall risk estimate for SPLC in Germany. Furthermore, we compared the results from the German analysis dataset to SEER data for the White population only to account for the majority Caucasian population in Germany.

### Software used

All analyses were conducted in R version 4.3.2 [22] with data management using the *tidyverse* and *tidytable* packages [23, 24]. Figures were created with *ggplot2* [25] and tables with *gt* [26]. The routines to calculate reference rates and stratified SIRs are publicly available in the R package *msSPChelpR* [27], and all scripts to create our analyses are published online [28].

## Results

### Description of the study population

Our analysis dataset comprised 135,572 German LC patients (31.8% females and 68.2% males) who survived for at least 6 months after LC diagnosis and fulfilled our inclusion criteria. The median age at initial diagnosis was 65.6 years in women and 67.2 years in men. Women were most frequently diagnosed with adenocarcinomas (44.8%) and small cell carcinomas (19.8%), while 34.5% of men had squamous cell carcinomas and 31.0% had adenocarcinomas. Patients had a mean follow-up of approximately 2.5 years. Over 70% of patients died after LC without developing another cancer, and another 24% were alive without an SPC event at the end of follow-up. In total, 154 women and 388 men (0.4% of patients) developed an SPLC. This corresponds to the crude incidence rate of 131.1 per 100,000 person-years in women and 168.4 in men. The other 4753 LC survivors (3.5%) developed a second cancer in a location other than the lung and bronchus (Table 1).

**Table 1:**
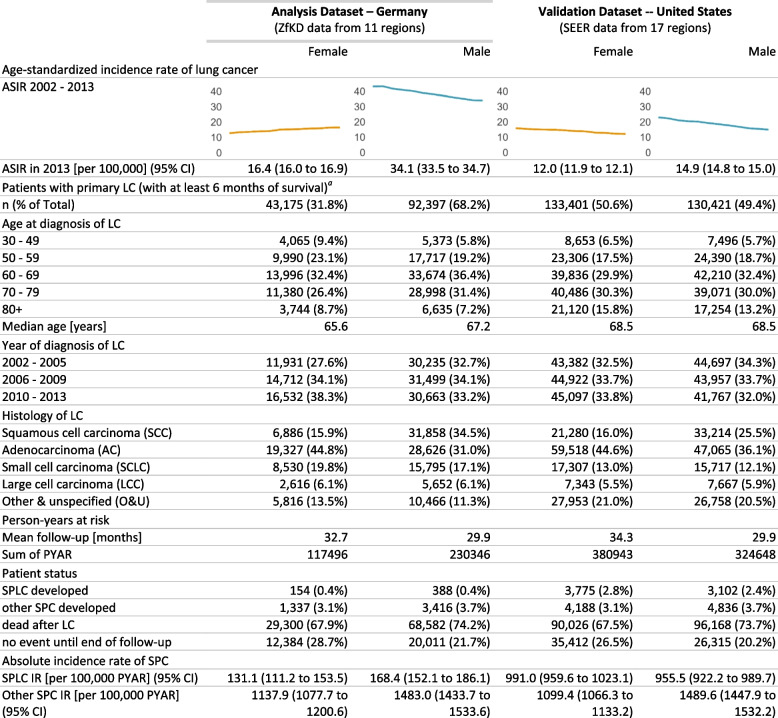
Characteristics of the analyzed study population with primary lung cancer. Age-standardized incidence rates of primary lung cancer (ASIR), follow-up time, characteristics of patients included in the main analysis with at least 6 months of survival, and absolute incidence of second primary cancer (SPC) by sex

*ASIR* Age-standardized incidence rate based on the World Standard Population 1960, *DCO* Death-certificate only, *IR* Incidence rate, *LC* Primary lung cancer, *PYAR* Person-years at risk, *SPC* Second primary cancer, *SPLC* Second primary lung cancer

^a^After exclusion by age and unusual histology

#### Evidence for bias using the conventional SIR estimates for same-site SPC

The most frequent histology groups of primary LC in our analysis dataset were adenocarcinomas (40.8% in women, 28.7% in men), squamous cell carcinomas (16.0% in women, 34.5% in men), and other specific carcinomas (34.1% in women, 27.9% in men) (Additional file 1: Fig. S7). Unspecified carcinomas and unspecified types of cancer comprised 8.8% of LC and, by IARC/IACR rules, could not result in the registration of an SPLC, even if one was diagnosed. In line with IARC/IACR registration rules, none diagnosed SPLC had the same histology group as the index LC in the German data. In contrast, the US validation data showed that the SEER registries had between 27.3 and 54.2% same histology group SPLC (Additional file 1: Table S8).

The simulation shows that real SIRs are underestimated when IARC/IACR rules are applied for SPLC. In the hypothetical case of SIR_real_ = 1.0, the simulated SIR_simIARC_ is 0.71 (95% CI 0.52–0.93) for women and 0.73 (95% CI 0.65–0.81) for men, incorrectly suggesting a risk reduction instead of no risk difference. A doubling of the risk in real SIR would translate to a SIR_simIARC_ of 1.41 for women and 1.45 for men. When simulating the US rates under IARC/IACR MP rules, we obtain a SIR_simIARC_ of 2.46 instead of SIR_real_ = 3.38 for men and a SIR_simIARC_ of 3.42 instead of SIR_real_ = 4.85 for women. Despite the differences in predominant histology groups of LC between men and women, we observed a similar underestimation of real SIR by approximately 30% for both sexes (Table 2).

**Table 2:**
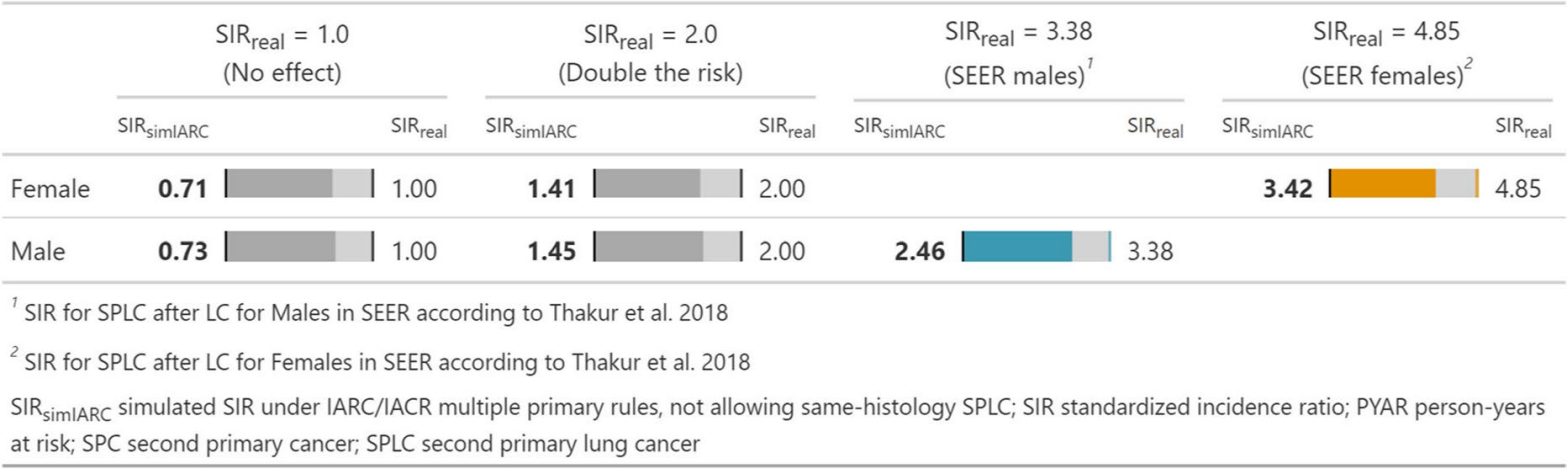
Estimating the risk for SPLC under IARC/IACR multiple primary rules SIR_simIARC_ given an assumed true risk SIR_real_

#### Risk estimates for SPLC after LC in Germany using histology-specific SIR

**Fig. 2:**
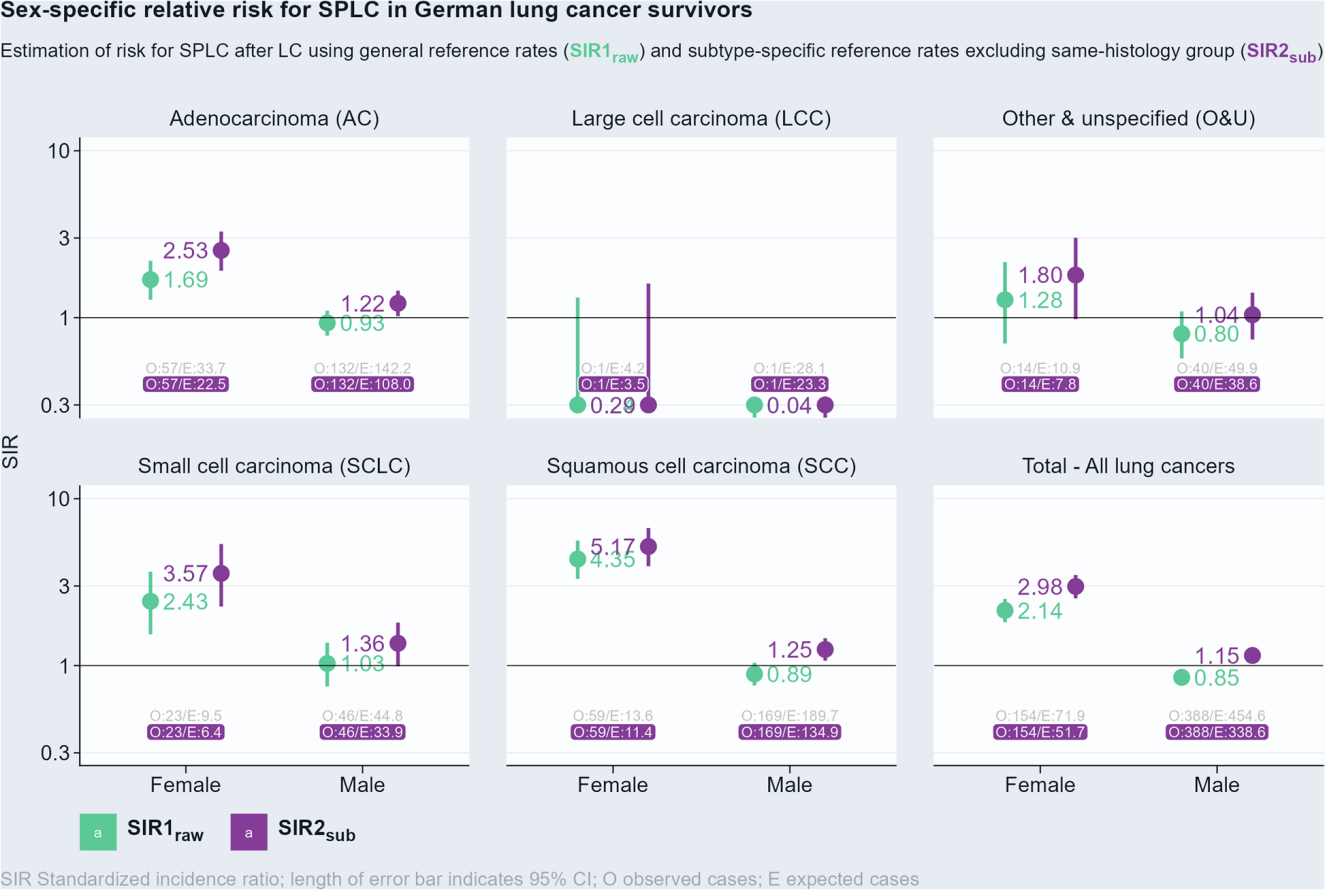
Sex-specific relative risk for SPLC in German lung cancer survivors. Estimation of risk for SPLC after LC using general reference rates (SIR1_raw_) shown in green and subtype-specific reference rates excluding same-histology group (SIR2_sub_) shown in purple. SIR estimates are presented stratified by histologic subtype (using the WHO classification of thoracic tumors) as well as a total of all histologies. Within each facet, SIR for females is shown on the left and for males on the right with the length of the line indicating the respective 95% CI. Observed and expected counts below in gray belong to the conventional method (SIR1_raw_) and those highlighted in purple yield the newly proposed risk estimate (SIR2_sub_)

The adjusted relative risk for LC survivors in Germany to develop an SPLC was 2.98 (95% CI 2.53–3.49) for females and 1.15 (95% CI 1.03–1.27) for males. The histological subtypes of index LC with the highest relative risk for SPLC were squamous cell carcinoma (SIR2_sub_ = 5.17, 95% CI 3.94–6.67) in women and SCLC (SIR2_sub_ = 1.36, 95% CI 0.99–1.81) in men. Figure 2 shows that the proposed method of using histological subtype-specific SIR resulted in higher risk estimates. This means that under this adjusted risk estimate, male LC survivors have a 15% higher risk for SPLC than the German male population. A risk increase was observed for all histological subtypes except for large-cell carcinoma. In contrast, the unadjusted SIR resulted in a 15% lower risk than in the male population (SIR1_raw_ = 0.85, 95% CI 0.77–0.94). For female LC survivors, the new method further increased risk estimates, particularly for adenocarcinoma (SIR2_sub_ = 2.53, 95% CI 1.91–3.28 vs. SIR1_raw_ = 1.69, 95% CI 1.28–2.19). When stratifying the analysis by time since LC diagnosis, the SIR2_sub_ increased with more prolonged survival for both sexes (Additional file 1: Fig. S9).

In the sensitivity analysis based on 75,272 person-years at risk from German PBCRs with low DCO rates, 106 SPLC occurred (19.6% of the initial analysis dataset). Both SIR1_raw_ and SIR2_sub_ were considerably lower than in the main analysis (SIR2_sub_ = 2.30 in females, SIR2_sub_ = 0.99 in males), but the overall pattern of substantial risk estimate increase using SIR2_sub_ remained (Additional file 1: Table S10).

#### Validation analysis using SEER data

Table 1 shows that our analysis dataset (German ZfKD data from 11 regions) and the validation dataset (US SEER data from 17 regions) are comparable concerning most characteristics of included LC survivors with similar age structure, histology group distribution, and patient status. However, Germany still has a growing age-standardized incidence rate for women, and the rate for men is at a higher level than in the USA. We also see that crude incidence rates for SPC in locations other than the lung and bronchus are similar for Germany (females 1137.9 and males 1483.0 per 100,000 person-years) and the USA (females 1099.4 and males 1489.6 per 100,000 person-years). The incidence of SPLC, however, differs greatly—the USA is showing 6 to 8 times higher crude rates than Germany (991.0 vs. 131.1 per 100,000 person-years for women and 955.5 vs. 168.4 for men).

The unadjusted risk estimates for SPLC in US LC survivors under SEER MP rules were more than twofold the rates under IARC/IACR MP rules (SIR1_raw_ = 5.52 and SIR3_IARC_ = 2.52 in women; SIR1_raw_ = 3.77 and SIR3_IARC_ = 1.71 in men). When using the histology-specific method, the risk estimates converge for overall SPLC risk (SIR2_sub_ = 4.37 and SIR4_subIARC_ = 3.58 in women; SIR2_sub_ = 2.94 and SIR4_subIARC_ = 2.34 in men) and for most subgroups presented in Table 3.

**Table 3:**
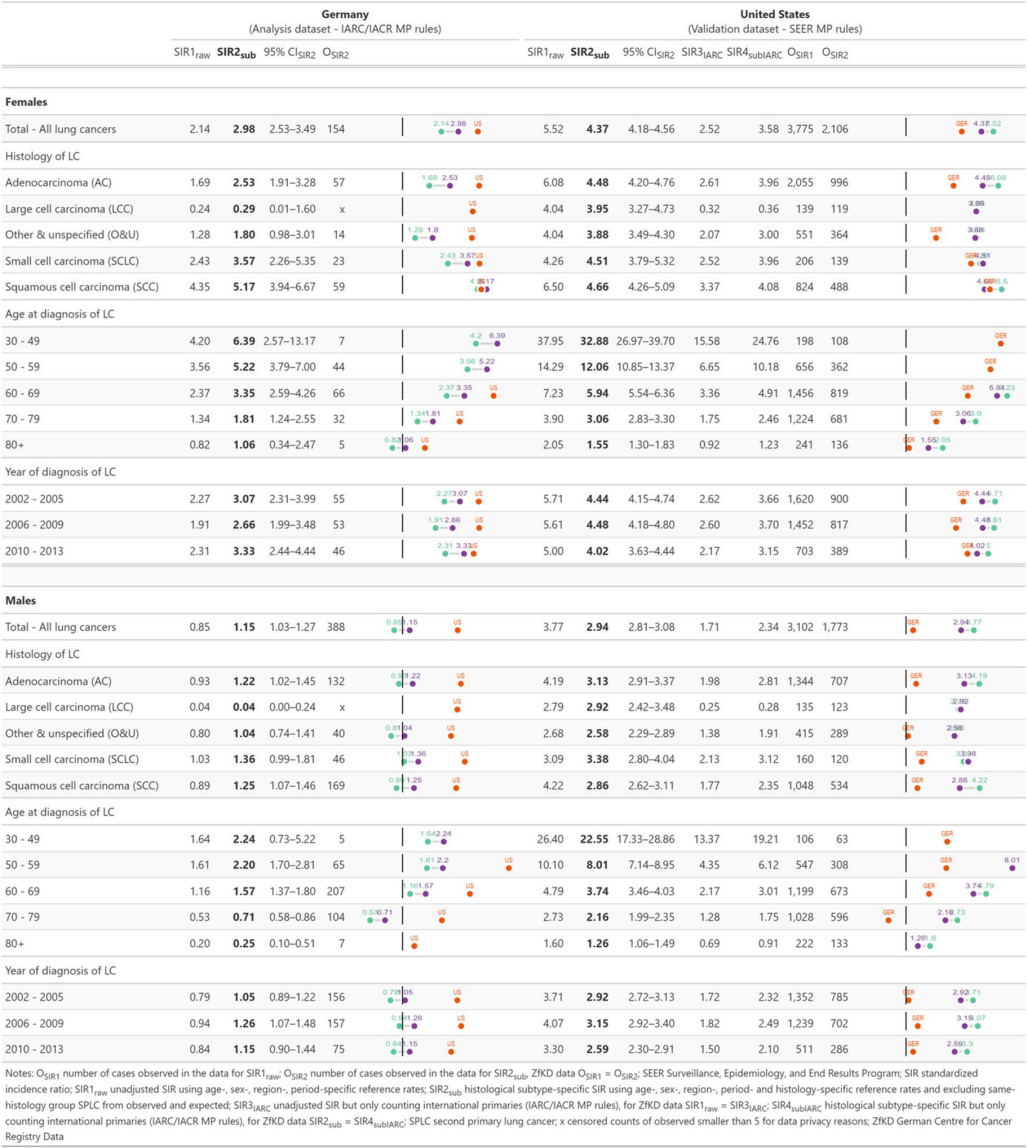
Validation analysis—risk for SPLC using unadjusted and histology-specific SIR method

Comparing estimates between Germany and the USA, we observed that SIR2_sub_ estimates are much closer than SIR1_raw_. In women, the gap narrows from 2.14 vs. 5.52 for unadjusted SIR to 2.98 vs. 4.37. In men, the wide unadjusted gap of 0.85 vs. 3.77 narrows slightly to 1.15 vs. 2.94. When examining the histology-specific estimate under IARC/IACR MP rules (SIR4_subIARC_), we still see a residual country difference with only slightly lower SPLC risk in German women (2.98 vs. 3.58) but half the risk for males compared to the USA (1.15 vs. 2.34). For some subgroups with a small number of observed cases in the German data, such as patients with large-cell carcinoma or below age 50, the risk gap between analysis and validation data may remain high, even after using the novel SIR estimate (Table 3).

The sensitivity analyses, comparing Germany to the White population in SEER, did not alter the results, showing that previous stratification of SIR by race adequately accounts for risk differences between races (Additional file 1: Table S11).

## Discussion

In this epidemiological study on the risk of SPLC, we demonstrated a bias introduced by the standard approach of calculating SIR for same-site SPC in cancer registries that use IARC/IACR MP rules. Our simulations showed an underestimation of true SIR by approximately 30% in both sexes because general population reference rates include tumors that cannot be observed in the at-risk population.

To minimize this bias, we proposed the method of histology-specific SIR and presented adjusted numbers to estimate the relative risk of SPLC in LC survivors in Germany. Using this method, the risk of being diagnosed with an SPLC was 2.98-fold for female LC survivors and 1.15-fold for male survivors compared to the general population. These numbers are substantially higher than previously published unadjusted SIR estimates using the standard approach (i.e., 2.06 and 0.83 for female and male LC survivors, respectively) [7].

Our validation analysis showed that the risk estimates for SPLC under SEER MP rules and the risk under IARC/IACR MP rules converge when using the proposed histology-specific compared to the standard approach. Moreover, the SPLC risk in Germany and the USA became comparable under the new method. In the descriptive analysis, we observed a similar incidence of primary LC, but an eight times higher crude incidence of SPLC in the USA compared to Germany, due to different registration rules and population structure. We narrowed this gap to an about twofold increased risk in the most comparable setting of histology-specific SIR counting international primaries only. Thus, we showed that the proposed histology-specific method makes the SIR metric more robust against differing or unclear MP rules and, therefore, more suitable for cross-country comparisons.

With the availability of long-term cancer registry data from SEER, relative risk estimates and survival analyses were predominantly published for the USA. Thakur et al. used 13 SEER registries for the period 1992 to 2007 and found a SIR estimate of 4.85 (4.66–5.05) in women and 3.38 (3.24–3.52) in men [9]. Our SIR1_raw_ estimates for a later period with more included regions were slightly higher for both sexes but—as expected for the same data source—showed very similar risk increases for younger age and adenocarcinoma index LC.

Other national population-based estimates of SPLC risk among LC survivors are sparse but include a study by Barclay et al. from the England cancer registry that showed an overall SIR for SPLC of 1.47 (not stratified by sex) for the same period, also using IARC/IACR MP rules and very similar case inclusion as our study [8]. They included almost 30% of unspecified LC diagnoses, which might explain their low overall unadjusted SIR estimate.

For Germany, we observed that using histology-specific calculation of risk for SPLC resulted in very different conclusions than the standard method. The previously published risk reduction for male LC survivors [7] turns into a risk increase in our updated analysis. Similarly, for the most frequent subtypes of LC, the risk of SPLC increases from a protective or null effect to a significant risk increase for LC survivors compared to the general population. In line with previous findings from Germany, the USA, and England, females have a higher relative risk for SPLC than males [7–9]. The results of bias simulation apply to other PBCRs using IARC/IACR MP rules because they depend only on the distribution of histology groups, which are similar across countries [29].

The SIR is an established measure in cancer epidemiology and beyond to identify at-risk populations and test etiologic hypotheses [19]. However, our analysis revealed the problem that the SIR metric can be seriously biased when observed and expected counts are based on different logics. Other examples are handling the intensified screening period of cancer survivors shortly after diagnosis [19], including DCO cases in the expected count [30], or stratifying by race.

A critical discussion in the analysis of SPC risk is the question of what constitutes biologically independent cancers. Although there might be a pathological answer, strict (IARC) and less stringent (SEER) rules for registering multiple primary cancers indicate different approaches. While IARC/IACR MP rules were historically focused on excluding potential metastases or recurrences, they give less importance to shared risk factors such as smoking, which can lead to preventable new cancers with the same appearance as the previous disease. Therefore, the SEER approach of registering SPC independent of histological differences and instead taking time into account has its merits. From the patient’s perspective, it is less relevant if, after a phase of complete remission, a new cancer of the same subtype occurs in the same site or whether this is an unexpected late recurrence. Both necessitate acute treatment according to the most recent guidelines and come with substantial psychological stress. From an epidemiological perspective, the differentiation between what constitutes a true SPC and late recurrence can be seen as arbitrary. Both are relevant to evaluating the disease risk of cancer survivors and play a role in the discussion about the need for increased surveillance. In particular, contralateral lung cancers are often underreported and instead seen as the spread of the original cancer [31]. On the other hand, it has been shown that certain multifocal tumors in some organs are likely to originate from the same transformed cells [32] and thus would not have been preventable. A potential solution to this dilemma could be to enrich existing PBCR with more information on metastases and recurrence and report the burden of both. Furthermore, improving the quality of cancer registries, e.g., increasing the share of microscopically verified cases, would reduce the role of unspecified LC and thus increase the precision of SPC differentiation under IARC/IACR MP rules.

This analysis also highlights the need to improve the clarity of IARC/IACR MP rules that have not been updated since 2004 and do not reflect improvements in (more fine-grained) histological classification since then. A simplified version using the broad LC categories recently published in the WHO Reporting System for Lung Cytopathology could be the common basis for a classification update [20, 33].

The importance of methodological choices on the validity of the SIR metric to determine and compare group-specific cancer risk has already been discussed. Crocetti et al. highlighted that we need to be clear about which cases to include in the observed count and the importance of applying the same logic when making cross-country comparisons and comparing extensively screened patients to the general population reference rates [34]. Our research extends this concern and highlights that including expected cases in the denominator of the SIR metric while these cases can never be observed—due to MP rules forbidding their registration—will introduce bias and result in an underestimation of cancer risk.

This study is the first nationwide analysis to provide reliable estimates of SPLC risk in German LC survivors. It adds to the sparse international numbers, as PBCR-based estimates have only been published for the USA and England. We newly developed the method of histological subtype-specific SIR that can reduce bias caused by using general population reference rates in determining the relative risk of same-site SPC for cancer registry data based on IARC/IACR MP rules. The method increases the comparability of SIR across countries when different MP rules are applied, information on registration practices is missing, or registration practices vary over time within a single PBCR. Furthermore, our study is the first to compare the relative risk of SPLC between Germany and the USA and provide estimates that allow SEER data to be used in international comparison.

Our proposed method has several limitations. In the situation in which PBCR data is incomplete, i.e., incident SPCs are not recorded in the registry, the proposed method still underestimates the SIR. This is especially relevant for IARC-compliant registries where SPCs are not recorded if the histology of the index tumor is unspecified. Moreover, the method works best for cancer entities that show a uniform distribution of incidence across histological subtypes. For example, the subtype is not informative in breast cancer, where adenocarcinoma comprises more than 90% of all malignancies. We encourage researchers with access to cancer registry data from other countries to test the proposed method. With regard to the estimation of SPLC risk for Germany, our study results are limited by varying completeness and quality of regional PBCR data—in particular, the existence of tumors without histological confirmation, a high share of DCO diagnoses, and incomplete follow-up of patients in cases of migration or partial record linkage with death registry data. Finally, our validation analysis also showed that for registry data under SEER MP rules, the histology-specific SIR generally suggests a lower risk than the unadjusted SIR (except primary SCLC). This could be an underestimation of risk if the true SIR of same-histology SPLC is higher than for different-histology tumors.

## Conclusions

In this study, we newly developed a method of histological subtype-specific SIR that can reduce bias when estimating the relative risk of same-site SPC. Our study shows that estimations for the relative risk of SPLC after LC are heavily influenced by differing registration practices for multiple primaries and how data is analyzed. Our approach of using histology-specific reference rates improves SIR estimations and facilitates cross-country comparison, especially for PBCRs that apply IARC/IACR MP rules. Future research can use this method to update epidemiological data, allowing clinicians to make informed decisions about patients’ SPLC risk based on accurate temporal and regional incidence trends. Differing registration practices remain an obstacle when comparing relative risk for SPC across countries and over time. Therefore, it should be further evaluated how MP rules and methods to determine SPC risk in the same location can be harmonized.

### Supplementary Information

**Additional file 1:** **Table S1.** Comparison of IARC/IACR and SEER multiple primary rules. **Table S2.** Details of dataset filtering. **Table S3.** Details of data modifications. **Table S4.** Data quality for included regions and SIR estimates. **Table S5.** Conversion table of histology codes into ICD-O-3 histologically ‘different’ groups and histological subtypes of lung cancer. **Section S6.** Details on simulations to estimate the size of bias using standard SIR. **Fig. S7.** Histological groups of LC and SPLC. **Table S8.** Frequency of same-histology SPLC by region. **Fig. S9.** Relative risk for SPLC in lung cancer survivors stratified by follow-up time. **Table S10.** Sensitivity analysis A – Risk of SPLC using unadjusted and histology-specific SIR method [restricted to six German PBCR with low DCO rate]. **Table S11.** Sensitivity analysis B – Risk of SPLC using unadjusted and histology-specific SIR method [SEER restricted to White population].

## Data Availability

Due to legal restrictions, the individual-level raw data used for this analysis (i.e., German cancer registry data) is only available via request to the German Centre for Cancer Registry Data (ZfKD), which can provide a scientific use file. More information on the application process is provided on the ZfKD website (https://www.krebsdaten.de/Krebs/EN/Content/ScientificUseFile/scientificusefile_node.html). The validation dataset (i.e., US cancer registry data) is publicly available via the Surveillance, Epidemiology, and End Results Program (SEER). More information on the data request process is provided on the SEER website (https://seer.cancer.gov/data/access.html). The authors of this paper strongly support open science and have therefore published both the analysis code and the newly programmed software to review and download under a GPL-3 license. The respective references are provided in the manuscript. Repository for analysis code: https://github.com/marianschmidt/pub_spc_sirmethods_bmed Repository for software code: https://doi.org/10.5281/zenodo.5055870.

## Abbreviations

CI: Confidence interval
E: Expected cases
IACR: International Association for Cancer Registration
IARC: International Agency for Research on Cancer
LC: Lung cancer
MP: Multiple primaries
O: Observed cases
PBCR: Population-based cancer registry
SEER: Surveillance, Epidemiology, and End Results Program
SIR: Standardized incidence ratio
SIR1_raw_: Unadjusted SIR using the conventional method
SIR2_sub_: Proposed new method using histology-specific reference rates to calculate SIR
SIR3_IARC_: Unadjusted SIR using the conventional method and only counting international primaries for both observed and expected cases
SIR4_subIARC_: Applying the new method of histology-specific SIR and only counting international primaries
SIR_real_: Assumed true SIR in reality
SIR_simIARC_: Simulated SIR when using international multiple primary rules
SPLC: Second primary lung cancer
ZfKD: German Centre for Cancer Registry Data

## References

[1] Sung H, Ferlay J, Siegel RL, Laversanne M, Soerjomataram I, Jemal A (2021). Global Cancer Statistics 2020: GLOBOCAN estimates of incidence and mortality worldwide for 36 cancers in 185 countries. CA Cancer J Clin.

[2] Zhou B, Zang R, Zhang M, Song P, Liu L, Bie F (2022). Worldwide burden and epidemiological trends of tracheal, bronchus, and lung cancer: a population-based study. EBioMedicine.

[3] Aredo JV, Luo SJ, Gardner RM, Sanyal N, Choi E, Hickey TP (2021). Tobacco smoking and risk of second primary lung cancer. J Thorac Oncol.

[4] Boakye EA, Buchanan P, Hinyard L, Osazuwa-Peters N, Simpson MC, Schootman M (2019). Trends in the risk and burden of second primary malignancy among survivors of smoking-related cancers in the United States. Int J Cancer.

[5] Park ER, Japuntich SJ, Rigotti NA, Traeger L, He Y, Wallace RB (2012). a snapshot of smokers following lung and colorectal cancer diagnosis. Cancer.

[6] Walker MS, Vidrine DJ, Gritz ER, Larsen RJ, Yan Y, Govindan R (2006). Smoking relapse during the first year after treatment for early-stage non-small-cell lung cancer. Cancer Epidemiol Biomarkers Prev.

[7] Eberl M, Tanaka LF, Kraywinkel K, Klug SJ (2022). Incidence of smoking-related second primary cancers after lung cancer in Germany: an analysis of nationwide cancer registry data. J Thorac Oncol.

[8] Barclay ME, Lyratzopoulos G, Walter FM, Jefferies S, Peake MD, Rintoul RC (2019). Incidence of second and higher order smoking-related primary cancers following lung cancer: a population-based cohort study. Thorax.

[9] Thakur MK, Ruterbusch JJ, Schwartz AG, Gadgeel SM, Beebe-Dimmer JL, Wozniak AJ (2018). Risk of second lung cancer in patients with previously treated lung cancer: analysis of Surveillance, Epidemiology, and End Results (SEER) data. J Thorac Oncol.

[10] IARC Working Group Report (2005). International rules for multiple primary cancers (ICD-O third edition). Eur J Cancer Prev.

[11] Fritz AG, Percy C, Jack A, Shanmugaratnam K, Sobin L, Parkin DM (2013). International classification of diseases for oncology: ICD-O (3rd edition, 1st revision).

[12] Johnson C, Peace S, Adamo P, Fritz A, Percy-Laurry A, Edwards BK. The 2007 multiple primary and histology coding rules. Bethesda, MD; 2007. https://seer.cancer.gov/tools/mphrules/download.html. Accessed 19 Apr 2024.

[13] Jégu J, Moitry M, Bara S, Trétarre B, Guizard A-V, Woronoff A-S (2017). Methodological issues of assessing the risk of a second cancer occurring in the same site as a first cancer using registry data. Cancer Epidemiol.

[14] Thun MJ, Linet MS, Cerhan JR, Haiman C, Schottenfeld D, editors. Cancer epidemiology and prevention. 4th ed. 2017.

[15] Sung H, Hyun N, Leach CR, Yabroff KR, Jemal A (2020). Association of first primary cancer with risk of subsequent primary cancer among survivors of adult-onset cancers in the United States. JAMA.

[16] Zentrum Für Krebsregisterdaten (ZfKD) Im Robert Koch-Institut. Datensatz des ZfKD auf Basis der epidemiologischen Landeskrebsregisterdaten Epi2016_2, verfügbare Diagnosejahre bis 2014. https://www.da-ra.de/dara/study/web_show?res_id=626806&mdlang=en&detail=true . 2017.

[17] Arndt V, Holleczek B, Kajüter H, Luttmann S, Nennecke A, Zeissig SR (2020). Data from population-based cancer registration for secondary data analysis: methodological challenges and perspectives. Gesundheitswesen.

[18] Surveillance, Epidemiology, and End Results (SEER) Program. SEER*Stat database: Incidence - SEER 9+13+18 regs merged research data + Hurricane Katrina impacted Louisiana cases, Nov 2018 sub (1975–2016 varying) linked to county attributes - time dependent (1990–2016) total U.S., 1969–2017 counties, released April 2019, based on the November 2018 submission. https://www.seer.cancer.gov.

[19] Curtis RE, Freedman DM, Ron E, Ries LAG, Hacker DG, Edwards BK, et al., editors. New malignancies among cancer survivors: SEER Cancer Registries, 1973–2000. Bethesda, MD: National Cancer Institute, NIR Publ. No. 05–5302; 2006.

[20] World Health Organization, IARC, editors. Thoracic tumours. 5th ed. Lyon: International Agency for Research on Cancer; 2021.

[21] Breslow NE, Day NE. Statistical methods in cancer research volume II: the design and analysis of cohort studies. Lyon: International Agency for Research on Cancer; 1987. 3329634

[22] R Core Team (2023). R: A language and environment for statistical computing.

[23] Wickham H, Averick M, Bryan J, Chang W, McGowan LD, François R (2019). Welcome to the Tidyverse. J Open Source Softw.

[24] Fairbanks M. Tidytable: tidy interface to ‘data.table’. 2020. https://CRAN.R-project.org/package=tidytable. Accessed 19 Apr 2024.

[25] Wickham H (2016). Ggplot2: elegant graphics for data analysis.

[26] Iannone R, Cheng J, Schloerke B. Gt: easily create presentation-ready display tables. 2020. https://CRAN.R-project.org/package=gt. Accessed 19 Apr 2024.

[27] Eberl M. msSPChelpR: helper functions for second primary cancer analyses. 2024. 10.5281/zenodo.4084961 . Accessed 19 Apr 2024.

[28] Eberl M. Code for Paper ‘Histology-specific standardized incidence ratio improves the estimation of second primary lung cancer risk’ - marianschmidt/pub_spc_sirmethods_bmed. 2023. https://github.com/marianschmidt/pub_spc_sirmethods_bmed. Accessed 19 Apr 2024.10.1186/s12916-024-03398-938702684

[29] Bray F, Colombet M, Mery L, Piñeros M, Znaor A, Zanetti R, et al., editors. Cancer incidence in five continents, Vol. XI (electronic version). Lyon: International Agency for Research on Cancer; 2021. https://publications.iarc.fr/Book-And-Report-Series/Iarc-Scientific-Publications/Cancer-Incidence-In-Five-Continents%C2%A0Volume-XI-2021. Accessed 19 Apr 2024.

[30] Eberl M, Tanaka LF (2020). Effect of counting DCO cases when calculating the relative incidence of second primary cancer (presentation).

[31] Johnson BE (1998). Second lung cancers in patients after treatment for an initial lung cancer. J Natl Cancer Inst.

[32] Werra C de, Donzelli I, Perone M, Micco RD, Orabona G. Multifocal and multicentric tumors. Renda A, editor. Milano: Springer Milan; 2009. p. 129–42.

[33] IAC-IARC-WHO Joint Editorial Board, editor. WHO Reporting System for Lung Cytopathology. 1st ed. Lyon: International Agency for Research on Cancer; 2022.

[34] Crocetti E, Buzzoni C, Giuliani O (2018). Methods for second primary cancer evaluation have to be standardized. Int J Cancer.

