# Supplementary material for: Histology-specific standardized incidence ratio improves the estimation of second primary lung cancer risk
Source: BMC Med. 2024 May 3;22:187. doi: 10.1186/s12916-024-03398-9 (PMC11069219; doi:10.1186/s12916-024-03398-9)
Supplement: Supplementary file 1 — Additional file 1: Table S1. Comparison of IARC/IACR and SEER multiple primary rules. Table S2. Details of dataset filtering. Table S3. Details of data modifications. Table S4. Data quality for included regions and SIR estimates. Table S5. Conversion table of histology codes into ICD-O-3 histologically ‘different’ groups and histological subtypes of lung cancer. Section S6. Details on simulations to estimate the size of bias using standard SIR. Fig. S7. Histological groups of LC and SPLC. Table S8. Frequency of same-histology SPLC by region. Fig. S9. Relative risk for SPLC in lung cancer survivors stratified by follow-up time. Table S10. Sensitivity analysis A – Risk of SPLC using unadjusted and histology-specific SIR method [restricted to six German PBCR with low DCO rate]. Table S11. Sensitivity analysis B – Risk of SPLC using unadjusted and histology-specific SIR method [SEER restricted to White population]. [file 12916_2024_3398_MOESM1_ESM.pdf]

Additional File 1 – Supplement S1-S11

Supplementary Information for the paper

"Histology-specific standardized incidence ratio improves the estimation of second primary lung cancer risk"

Authors: Eberl M, Tanaka LF, Kraywinkel K, Klug SJ

Correspondence: Stefanie Klug, stefanie.klug@tum.de, Chair of Epidemiology, TUM Department of Sport and Health Sciences, Technical University of Munich, Munich, Germany

Table S1: Comparison of IARC/IACR and SEER multiple primary rules ... 2

Table S2: Details of dataset filtering ... 3

Table S3: Details of data modifications ... 4

Table S4: Data quality for included regions and SIR estimates ... 5

Table S5: Conversion table of histology codes into ICD-O-3 histologically 'different' groups and histological subtypes of lung cancer ... 6

Section S6: Details on simulations to estimate the size of bias using standard SIR ... 7

Figure S7: Histological groups of LC and SPLC ... 8

Table S8: Frequency of same-histology SPLC by region ... 9

Figure S9: Relative risk for SPLC in lung cancer survivors stratified by follow-up time ... 10

Table S10: Sensitivity analysis A – Risk of SPLC using unadjusted and histology-specific SIR method [restricted to six German PBCR with low DCO rate] ... 11

Table S11: Sensitivity analysis B – Risk of SPLC using unadjusted and histology-specific SIR method [SEER restricted to White population] ... 12

Table S1: Comparison of IARC/IACR and SEER multiple primary rules

Table S1: Comparison of IARC/IACR and SEER multiple primary rules

	IARC/IACR MP rules	SEER MP rules	Expected incidence of SPLC
**Timing**	irrelevant	• after 1-5 “disease-free” years [3 years for lung cancer] the exact same cancer (location, behavior, histology, laterality) will be recorded as ✅ SPC<br>• after 60 days the same cancer (location, histology, laterality) with different behavior will be recorded as ✅ SPC	higher SPLC incidence for SEER MP rules
**Location**	• different organ: ✅ SPC<br>• same organ: ❌ no SPC	different location (mostly at third topography character, i.e. C33 is different from C34): ✅ SPC	higher SPLC incidence for SEER MP rules
**Laterality** (different side in same location)	❌ no SPC	different side: ✅ SPC	higher SPLC incidence for SEER MP rules
**Histology** (for same location and laterality)	• different histological group (wide groups): ✅ SPC<br>• unknown or unspecified histology: ❌ no SPC	• histology differs in third digit xxxx (narrow groups): ✅ SPC<br>• carcinoma/sarcoma NOS follows specified carcinoma/sarcoma or vice versa: ❌ no SPC	higher SPLC incidence for SEER MP rules
**Behavior** (for same location, laterality and histology)	❌ no SPC	more than 60 days in between cancers: ✅ SPC	not relevant, because study only takes into account malignant behavior

IARC/IACR multiple primary rules according to [10] IARC Working Group Report. International rules for multiple primary cancers (ICD-O third edition). European Journal of Cancer Prevention 2005;14:307–8.

SEER multiple primary rules according to [12] Johnson C, Peace S, Adamo P, Fritz A, Percy-Laurry A, Edwards BK. The 2007 Multiple Primary and Histology Coding Rules. Bethesda, MD: National Cancer Institute, SEER; 2007.

Table S2: Details of dataset filtering

Table S2: Details of dataset filtering

Filtering Stage	N excluded	N remaining	Code	Comments
**Germany (Analysis dataset - ZfKD)**				
0 – all patients with a first cancer diagnosis of lung cancer in raw data	NA	705714	`filter(t_sitewhogen.1 == "Lung and Bronchus")`	has been pre-filtered according to the following criteria:<br>• patients with a first cancer diagnosis of lung cancer defined as:<br>○ ICD-10-GM code `C34` AND<br>○ `t_tumid == 1` (Tumour ID created with `msSPChelpR::renumber_time_id()` function and defined as chronologically sorting of all registered cases per patient ID by date of diagnosis, ignoring D diagnoses [in situ, benign and unknown behaviour diagnoses] and C44 diagnoses [non-malignant melanoma])<br>• no further restrictions on age, registry, site, histology or data quality
1 – only select LC diagnoses 2002-–2013	217826	487888	`filter((t_datediag.1 > "2002-01-01" & t_datediag.1 < "2013-12-31"))`	Date of diagnosis is only recorded with month and year of diagnosis (variable name in source data was DDIMP); in order to calculate with dates the day of diagnosis is always set to the 15th of the month
2 – only select registers with acceptable data quality	223490	264398	`filter(p_region.1 %in% c("DE2 Bavaria", "DE4 Brandenburg", "DE5 Bremen", "DE6 Hamburg","DE8 Mecklenburg-Western Pomerania", "DE9 Lower Saxony", "DEA3 Muenster", "DEC Saarland", "DED Saxony", "DEF Schleswig-Holstein", "DEG Thuringia"))`	Selection critieria: folow-up of at least 5 years, covering at least 90% of incident cancer cases in the registry area as recommended by the Association of PBCR in Germany[1]
3 – only select LC cases diagnosed at age 30-–99	285	264113	`filter(t_agediag.1 >= 30 & t_agediag.1 < 100)`	
4 – delete cases with unusual LC morphology	179	263934	`filter(!(t_sublungiarc.1 %in% c("Excluded", "Unusual")) & !(t_sublung.1 %in% c("Excluded - unusual")))`	Variable `t_sublungiarc.1` was created from histology codes according to Supplement Table "Definition of histological subtypes of lung cancer".
5 – delete cases where LC is DCO	38914	225020	`filter(t_confirm.1 != "DCO" | is.na(t_confirm.1))`	Case confirmation was death certificate only (variable in source data was DSICH).Cases where source of diagnosis is missing are not removed.
6 – delete cases with less than 6 months follow-up	89447	135573	`filter(p_futimeyrs >= 0.5)`	Minimum follow-up without SPC, death or end of FU >= 6 months (0.5 years) after first lung cancer diagnosis. Cases with missing follow-up time are also excluded.
7 – delete cases with unusual SPLC morphology	1	135572	`filter(!(t_sublungiarc.2 %in% c("Excluded", "Unusual")) & !(t_sublung.2 %in% c("Excluded - unusual")))`	Variable `t_sublungiarc.2` was created from histology codes according to Supplement Table "Definition of histological subtypes of lung cancer".
**United States (Verification dataset - SEER)**				
0 – all patients with a first cancer diagnosis of lung cancer in raw data	NA	1079373	`filter(t_sitewhogen.1 == "Lung and Bronchus")`	has been pre-filtered according to the following criteria:<br>• patients with a first cancer diagnosis of lung cancer defined as:<br>○ SITERWHO code `39` (Lung and Bronchus) AND<br>○ `t_tumid == 1` (Tumour ID created with `msSPChelpR::renumber_time_id()` function and defined as chronologically sorting of all registered cases per patient ID by date of diagnosis, excluding non-malignant tumors benign, borderline, in situ; use BEHO3V variable as of SEER 2022 Standard) and Other Non-Epithelial Skin cancer (sitewho=45), since this is excluded from most international summary counts and also for German analysis data no refrates are available)<br>• no further restrictions on age, registry, site, histology or data quality
1 – only select LC diagnoses 2002-–2013	558347	521026	`filter((t_datediag.1 > "2002-01-01" & t_datediag.1 < "2013-12-31"))`	Date of diagnosis is only recorded with month and year of diagnosis (variable name in source data was YEAR_DX for year and MDXRECMP for month); in order to calculate with dates the day of diagnosis is always set to the 15th of the month
2 – only select registers with acceptable data quality	37150	483876	`filter(p_region.1 %in% c("SEER Reg 01 - San Francisco-Oakland SMSA", "SEER Reg 02 - Connecticut", "SEER Reg 20 - Detroit (Metropolitan)", "SEER Reg 21 - Hawaii", "SEER Reg 22 - Iowa", "SEER Reg 23 - New Mexico", "SEER Reg 25 - Seattle (Puget Sound)", "SEER Reg 26 - Utah", "SEER Reg 27 - Atlanta (Metropolitan)", "SEER Reg 29 - Alaska Natives", "SEER Reg 31 - San Jose-Monterey", "SEER Reg 35 - Los Angeles", "SEER Reg 37 - Rural Georgia", "SEER Reg 41 - California excluding SF/SJM/LA", "SEER Reg 42 - Kentucky", "SEER Reg 44 - New Jersey", "SEER Reg 47 - Georgia excluding Atlanta/Rural Georgia"))`	Selection critieria:all registries that started before 2002 (SEER-18) excluding Louisiana (Hurricane Katrina impact)
3 – only select LC cases diagnosed at age 30-–99	954	482922	`filter(t_agediag.1 >= 30 & t_agediag.1 < 100)`	
4 – delete cases with unusual LC morphology	293	482629	`filter(!(t_sublungiarc.1 %in% c("Excluded", "Unusual")) & !(t_sublung.1 %in% c("Excluded - unusual")))`	Variable `t_sublungiarc.1` was created from histology codes according to Supplement Table "Definition of histological subtypes of lung cancer".
5 – delete cases where LC is DCO	1773	480856	`filter(t_confirm.1 != "DCO" | is.na(t_confirm.1))`	Case confirmation was death certificate only (variable in source data was REPT_SRC).Cases where source of diagnosis is missing are not removed.
6 – delete cases with less than 6 months follow-up	217031	263825	`filter(p_futimeyrs >= 0.5)`	Minimum follow-up without SPC, death or end of FU >= 6 months (0.5 years) after first lung cancer diagnosis. Cases with missing follow-up time are also excluded.
7 – delete cases with unusual SPLC morphology	3	263822	`filter(!(t_sublungiarc.2 %in% c("Excluded", "Unusual")) & !(t_sublung.2 %in% c("Excluded - unusual")))`	Variable `t_sublungiarc.2` was created from histology codes according to Supplement Table "Definition of histological subtypes of lung cancer".

[1] GEKID. Atlas der Krebsinzidenz und Krebsmortalität der Gesellschaft der epidemiologischen Krebsregister in Deutschland e.V. (GEKID) [Internet]. Lübeck: Gesellschaft der epidemiologischen Krebsregister in Deutschland e.V.; 2021 [cited 2023 Jun 30] p. 20. Available from: https://atlas.gekid.de/CurrentVersion/Methoden%20GEKID%20Atlas.pdf

Table S3: Details of data modifications

Table S3: Details of data modifications

Variable	Detailed description	Code
Patient Status at end of follow-up<br>[p_status]	• using pat_status_tt function from msSPChelpR package<br>• set end of follow-up time to end of December 2014 (follow-up data for new cancer cases is not available after that data)<br>• impute information for missing date of death by taking the last available date for all patients that are dead according to available life status (→ for 901 cases DOD was imputed by using last available date of diagnosis, thus assuming 0 survival time)	msSPChelpR::pat_status_tt(<br>fu_end = "2014-12-15", dattype = NULL, status_var = "p_status", life_var = "p_dead.1", spc_var = "p_spc", birthdat_var = "p_datebirth", lifedat_var = "p_datedeath", lifedatmin_var = "p_dodmin.1", fcdat_var = "t_datediag.1", spcdat_var = "t_datediag.2", life_stat_alive = "alive", life_stat_dead = "dead", spc_stat_yes = "SPC developed", spc_stat_no = "No SPC", lifedat_fu_end = NULL, use_lifedatmin = TRUE, check = TRUE, as_labelled_factor = TRUE<br>)
Follow-up time of patient in years<br>[p_futimeyrs]	• using calc_futime_tt function from msSPChelpR package<br>• follow-up time of patient from diagnosis of first cancer until SPC or date of death death or end of FU [years]<br>• set end of follow-up time to end of December 2014 (follow-up data for new cancer cases is not available after that data)<br>• for all cases where patient status at end of follow-up period cannot be determined (p_status equals 97, 98 or 99) the follow-up time is set to missing	msSPChelpR::calc_futime_tt(<br>futime_var_new = "p_futimeyrs", fu_end = "2014-12-15", dattype = NULL, time_unit = "years", status_var = "p_status", lifedat_var = "p_datedeath", fcdat_var = "t_datediag.1", spcdat_var = "t_datediag.2"<br>)
Type of diagnostic confirmation<br>[t_confirm]	• aggregate available information to categories 1=autopsy 2=clinical (without diagnostics) 3=clinical with diagnostics 4=cytology 5=DCO 6=histology 7=tumor markers 99=unknown	ZfKD data:<br>t_confirm = case_when(DSICH == "autopsy" ~ "autopsy", DSICH == "DCO (death certificate only)" ~ "DCO", DSICH == "histology of metastasis" ~ "histology", DSICH == "histology of primary tumor" ~ "histology", DSICH == "other diagnosis" ~ "unknown", TRUE ~ as.character(DSICH))<br><br>SEER data:<br>t_confirm = case_when(REPT_SRC == "Autopsy Only" ~ "autopsy", REPT_SRC == "Death Certificate Only)" ~ "DCO", DX_CONF == "Positive histology" ~ "histology", DX_CONF == "Positive exfoliative cytology, no positive histology" ~ "cytology", DX_CONF == "Positive histology PLUS - positive immunophenotyping AND/OR positive genetic studies (Used only for hematopoietic and lymphoid neoplasms M-9590/3-9992/3)" ~ "histology", DX_CONF == "Positive laboratory test/marker study" ~ "tumor markers", DX_CONF == "Positive microscopic confirmation, method not specified" ~ "clinical with diagnostics", DX_CONF == "Direct visualization without microscopic confirmation" ~ "clinical with diagnostics", DX_CONF == "Radiology and other imaging techniques without microscopic confirmation" ~ "clinical with diagnostics", DX_CONF == "Clinical diagnosis only (other than 5, 6, or 7)" ~ "clinical (without diagnostics)", DX_CONF == "Unknown whether microscopically confirmed; death certificate only" & p_datedeath == t_datediag ~ "DCO", DX_CONF == "Unknown whether microscopically confirmed; death certificate only" ~ "unknown", TRUE ~ as.character(DX_CONF))
Groups of malignant neoplasms histologically different<br>[t_histgroupiarc]	• using histgroup_iarc function from msSPChelpR package<br>• based on 4-digit morphology code in variable 't_hist'<br>• using classification in ICD-O-3 revision 1, released 2013	histgroup_iarc(hist_var = t_hist, new_var_hist = t_histgroupiarc, version = "3.1")

Table S4: Data quality for included regions and SIR estimates

Table S4: Data quality for included regions and SIR estimates

Regional Registry	LC Cases	Type of diagnostic confirmation for all LC: DCO / Microscopic / Other [1]	PYAR	Cases all SPC (%)	Cases SPLC (%)	Risk for SPLC - SIR (95% CI): $SIR1_{raw}$ Female	$SIR2_{sub}$ Female	$SIR1_{raw}$ Male	$SIR2_{sub}$ Male
**Germany - ZfKD**									
DE2 Bavaria	33,340	21% / 76% / 3%	88,402	1347 (4.0%)	87 (0.3%)	1.34 (0.84—2.03)	1.86 (1.17—2.82)	0.76 (0.59—0.97)	1 (0.77—1.28)
DE4 Brandenburg	9,736	0.9% / 81.5% / 7.6%	24,193	304 (3.1%)	67 (0.7%)	4.29 (2.5—6.86)	5.85 (3.41—9.37)	1.38 (1.02—1.81)	1.84 (1.37—2.43)
DE5 Bremen	3,217	.20% / 85.31% / 7.49%	9,041	135 (4.2%)	18 (0.6%)	1.56 (0.51—3.64)	2.27 (0.74—5.29)	0.88 (0.47—1.5)	1.22 (0.65—2.09)
DE6 Hamburg	6,929	.9% / 84.6% / 6.5%	17,525	305 (4.4%)	7 (0.1%)	0.46 (0.1—1.35)	0.65 (0.13—1.89)	0.17 (0.05—0.44)	0.23 (0.06—0.59)
DE8 Mecklenburg-Western Pomerania	6,536	0.4% / 82.7% / 6.9%	15,615	224 (3.4%)	32 (0.5%)	2.62 (1.06—5.41)	3.64 (1.46—7.5)	1 (0.65—1.48)	1.34 (0.87—1.98)
DE9 Lower Saxony	28,736	15.3% / 65.8% / 18.9%	72,266	1231 (4.3%)	53 (0.2%)	1.01 (0.56—1.66)	1.41 (0.79—2.33)	0.4 (0.28—0.55)	0.54 (0.38—0.74)
DEA3 Muenster	10,011	12.6% / 78.0% / 9.4%	25,068	400 (4.0%)	77 (0.8%)	4.68 (3.09—6.81)	6.47 (4.26—9.41)	1.27 (0.94—1.67)	1.71 (1.27—2.25)
DEC Saarland	4,737	9.0% / 83.4% / 7.6%	12,844	119 (2.5%)	0	0 (0—1.16)	0 (0—1.61)	0 (0—0.17)	0 (0—0.23)
DED Saxony	13,544	.4% / 81.7% / 10.0%	34,304	464 (3.4%)	61 (0.5%)	2.64 (1.32—4.73)	3.66 (1.83—6.55)	1.03 (0.76—1.36)	1.39 (1.03—1.83)
DEF Schleswig-Holstein	11,404	19% / 77% / 4%	29,085	531 (4.7%)	97 (0.9%)	4.43 (3.15—6.06)	6.11 (4.35—8.35)	1.52 (1.16—1.97)	2.04 (1.55—2.63)
DEG Thuringia	7,382	13% / 82% / 5%	19,498	235 (3.2%)	43 (0.6%)	3.36 (1.45—6.63)	4.65 (2.01—9.16)	1.25 (0.87—1.74)	1.72 (1.2—2.39)
**United States - SEER**									
SEER Reg 01 - San Francisco-Oakland SMSA	11,863	% / 87% / 12%	32,582	592 (5.0%)	240 (2.0%)	4.69 (3.92—5.57)	3.94 (3.09—4.94)	3.75 (3.08—4.52)	2.87 (2.19—3.7)
SEER Reg 02 - Connecticut	14,517	2% / 90.4% / 9.4%	41,376	983 (6.8%)	460 (3.2%)	5.9 (5.22—6.65)	5.1 (4.34—5.96)	4.24 (3.66—4.89)	3.5 (2.88—4.21)
SEER Reg 20 - Detroit (Metropolitan)	16,852	% / 89% / 11%	45,482	1175 (7.0%)	511 (3.0%)	5.49 (4.89—6.15)	4.14 (3.52—4.83)	3.61 (3.14—4.12)	2.68 (2.21—3.21)
SEER Reg 21 - Hawaii	4,170	2% / 89.8% / 10.0%	11,512	207 (5.0%)	91 (2.2%)	5.96 (4.33—8)	4.52 (2.83—6.84)	3.91 (2.87—5.2)	2.82 (1.8—4.19)
SEER Reg 22 - Iowa	12,194	% / 86% / 13%	31,027	776 (6.4%)	349 (2.9%)	5.91 (5.03—6.89)	5.24 (4.27—6.36)	4.59 (3.96—5.3)	3.82 (3.15—4.59)
SEER Reg 23 - New Mexico	4,730	% / 81% / 18%	12,325	169 (3.6%)	65 (1.4%)	3.19 (2.12—4.61)	2.23 (1.22—3.75)	3.53 (2.49—4.87)	2.56 (1.57—3.96)
SEER Reg 25 - Seattle (Puget Sound)	14,891	% / 87% / 13%	39,455	985 (6.6%)	429 (2.9%)	5.89 (5.18—6.68)	4.03 (3.34—4.83)	4.35 (3.74—5.02)	3.93 (3.26—4.69)
SEER Reg 26 - Utah	2,929	% / 87% / 13%	7,665	139 (4.7%)	48 (1.6%)	8.24 (5.22—12.36)	5.15 (2.47—9.48)	6.18 (4—9.12)	4.01 (2.07—7)
SEER Reg 27 - Atlanta (Metropolitan)	7,989	% / 90.7% / 9.2%	21,413	479 (6.0%)	218 (2.7%)	6.76 (5.61—8.07)	5.42 (4.21—6.85)	4.57 (3.7—5.58)	3.34 (2.5—4.39)
SEER Reg 29 - Alaska Natives	373	% / 86% / 14%	905	12 (3.2%)	8 (2.1%)	6.4 (2.08—14.93)	3.7 (0.45—13.38)	2.97 (0.61—8.68)	4.37 (0.9—12.78)
SEER Reg 31 - San Jose-Monterey	5,430	% / 89% / 11%	15,129	285 (5.2%)	104 (1.9%)	5.8 (4.53—7.32)	3.88 (2.65—5.48)	2.89 (1.99—4.06)	2.47 (1.51—3.82)
SEER Reg 35 - Los Angeles	20,707	% / 89% / 11%	57,262	1137 (5.5%)	465 (2.2%)	6.34 (5.61—7.15)	4.85 (4.08—5.71)	4 (3.46—4.6)	3.05 (2.51—3.68)
SEER Reg 37 - Rural Georgia	633	% / 86% / 14%	1,549	35 (5.5%)	11 (1.7%)	5.03 (1.84—10.94)	6.06 (1.97—14.15)	2.22 (0.72—5.18)	1.86 (0.38—5.45)
SEER Reg 41 - California excluding SF/SJM/LA	57,612	% / 87% / 13%	153,812	3236 (5.6%)	1291 (2.2%)	4.79 (4.45—5.15)	3.62 (3.27—4)	3.63 (3.34—3.95)	2.58 (2.29—2.89)
SEER Reg 42 - Kentucky	26,986	% / 86% / 13%	67,443	1816 (6.7%)	916 (3.4%)	5.94 (5.41—6.5)	4.74 (4.19—5.34)	3.61 (3.28—3.95)	2.78 (2.45—3.14)
SEER Reg 44 - New Jersey	35,900	% / 89% / 11%	101,006	2327 (6.5%)	973 (2.7%)	5.31 (4.88—5.76)	4.57 (4.1—5.08)	3.67 (3.32—4.05)	3.07 (2.7—3.48)
SEER Reg 47 - Georgia excluding Atlanta/Rural Georgia	26,046	% / 87% / 12%	65,650	1548 (5.9%)	698 (2.7%)	6.12 (5.49—6.8)	4.82 (4.17—5.54)	3.38 (3.04—3.75)	2.71 (2.36—3.1)

[1] Microscopic diagnoses include cytology, and histology of the tumor. Other diagnoses include autopsy, clinical (without diagnostics), clinical with diagnostics, tumor markers and missing information on source of diagnosis.

SIR Standardized incidence ratio; DCO death-certificate only; LC primary lung cancer; PYAR person-years at risk; SPC second primary cancer; SPLC second primary lung cancer

Table S5: Conversion table of histology codes into ICD-O-3 histologically ‘different’ groups and histological subtypes of lung cancer

Table S5: Conversion table of histology codes into ICD-O-3 histologically 'different' groups and histological subtypes of lung cancer

**Histology Code**	**Groups histologically ‘different’** (ICD-O-3 rev 1)	**Histological type of lung cancer** (IARC classification)	**Histology Code**	**Groups histologically ‘different’** (ICD-O-3 rev 1)	**Histological type of lung cancer** (IARC classification)	**Histology Code**	**Groups histologically ‘different’** (ICD-O-3 rev 1)	**Histological type of lung cancer** (IARC classification)	**Histology Code**	**Groups histologically ‘different’** (ICD-O-3 rev 1)	**Histological type of lung cancer** (IARC classification)
8000	Unspecified types of cancer	Other & unspecified (O&U)	8144	Adenocarcinomas	Other & unspecified (O&U)	8503	Adenocarcinomas	Other & unspecified (O&U)	8901	Sarcomas and soft tissue tumours	Other & unspecified (O&U)
8001	Unspecified types of cancer	Other & unspecified (O&U)	8145	Adenocarcinomas	Other & unspecified (O&U)	8507	Adenocarcinomas	Other & unspecified (O&U)	8902	Sarcomas and soft tissue tumours	Other & unspecified (O&U)
8002	Unspecified types of cancer	Other & unspecified (O&U)	8147	Adenocarcinomas	Other & unspecified (O&U)	8525	Adenocarcinomas	Other & unspecified (O&U)	8910	Sarcomas and soft tissue tumours	Other & unspecified (O&U)
8003	Unspecified types of cancer	Other & unspecified (O&U)	8154	Other specific carcinomas	Other & unspecified (O&U)	8530	Adenocarcinomas	Other & unspecified (O&U)	8912	Sarcomas and soft tissue tumours	Other & unspecified (O&U)
8004	Unspecified types of cancer	Other & unspecified (O&U)	8170	Other specific carcinomas	Other & unspecified (O&U)	8550	Adenocarcinomas	Adenocarcinoma (AC)	8920	Sarcomas and soft tissue tumours	Other & unspecified (O&U)
8005	Unspecified types of cancer	Other & unspecified (O&U)	8190	Adenocarcinomas	Other & unspecified (O&U)	8551	Adenocarcinomas	Adenocarcinoma (AC)	8921	Sarcomas and soft tissue tumours	Other & unspecified (O&U)
8010	Unspecified carcinomas (NOS)	Large cell carcinoma (LCC)	8200	Adenocarcinomas	Other & unspecified (O&U)	8560	Other specific carcinomas	Other & unspecified (O&U)	8933	Other specified types of cancer	Unusual
8011	Unspecified carcinomas (NOS)	Large cell carcinoma (LCC)	8201	Adenocarcinomas	Other & unspecified (O&U)	8562	Other specific carcinomas	Other & unspecified (O&U)	8935	Other specified types of cancer	Other & unspecified (O&U)
8012	Unspecified carcinomas (NOS)	Large cell carcinoma (LCC)	8210	Adenocarcinomas	Other & unspecified (O&U)	8570	Adenocarcinomas	Adenocarcinoma (AC)	8940	Adenocarcinomas	Unusual
8013	Unspecified carcinomas (NOS)	Other & unspecified (O&U)	8211	Adenocarcinomas	Adenocarcinoma (AC)	8571	Adenocarcinomas	Adenocarcinoma (AC)	8941	Adenocarcinomas	Other & unspecified (O&U)
8014	Unspecified carcinomas (NOS)	Large cell carcinoma (LCC)	8230	Other specific carcinomas	Adenocarcinoma (AC)	8572	Adenocarcinomas	Adenocarcinoma (AC)	8951	Other specified types of cancer	Other & unspecified (O&U)
8015	Unspecified carcinomas (NOS)	Large cell carcinoma (LCC)	8231	Other specific carcinomas	Adenocarcinoma (AC)	8574	Adenocarcinomas	Adenocarcinoma (AC)	8963	Other specified types of cancer	Unusual
8020	Unspecified carcinomas (NOS)	Large cell carcinoma (LCC)	8240	Other specific carcinomas	Other & unspecified (O&U)	8575	Adenocarcinomas	Other & unspecified (O&U)	8972	Other specified types of cancer	Other & unspecified (O&U)
8021	Unspecified carcinomas (NOS)	Large cell carcinoma (LCC)	8241	Other specific carcinomas	Other & unspecified (O&U)	8576	Adenocarcinomas	Adenocarcinoma (AC)	8973	Other specified types of cancer	Other & unspecified (O&U)
8022	Unspecified carcinomas (NOS)	Large cell carcinoma (LCC)	8243	Other specific carcinomas	Other & unspecified (O&U)	8580	Other specific carcinomas	Other & unspecified (O&U)	8980	Other specified types of cancer	Other & unspecified (O&U)
8030	Other specific carcinomas	Large cell carcinoma (LCC)	8244	Other specific carcinomas	Other & unspecified (O&U)	8581	Other specific carcinomas	Unusual	8982	Other specified types of cancer	Other & unspecified (O&U)
8031	Other specific carcinomas	Large cell carcinoma (LCC)	8245	Other specific carcinomas	Other & unspecified (O&U)	8585	Other specific carcinomas	Other & unspecified (O&U)	8990	Sarcomas and soft tissue tumours	Other & unspecified (O&U)
8032	Other specific carcinomas	Other & unspecified (O&U)	8246	Other specific carcinomas	Other & unspecified (O&U)	8680	Sarcomas and soft tissue tumours	Other & unspecified (O&U)	9015	Other specified types of cancer	Other & unspecified (O&U)
8033	Other specific carcinomas	Other & unspecified (O&U)	8247	Other specific carcinomas	Other & unspecified (O&U)	8710	Sarcomas and soft tissue tumours	Other & unspecified (O&U)	9040	Sarcomas and soft tissue tumours	Other & unspecified (O&U)
8034	Other specific carcinomas	Other & unspecified (O&U)	8249	Other specific carcinomas	Other & unspecified (O&U)	8711	Sarcomas and soft tissue tumours	Other & unspecified (O&U)	9041	Sarcomas and soft tissue tumours	Other & unspecified (O&U)
8035	Other specific carcinomas	Large cell carcinoma (LCC)	8250	Other specific carcinomas	Adenocarcinoma (AC)	8720	Other specified types of cancer	Other & unspecified (O&U)	9043	Sarcomas and soft tissue tumours	Other & unspecified (O&U)
8040	Other specific carcinomas	Other & unspecified (O&U)	8251	Other specific carcinomas	Adenocarcinoma (AC)	8743	Other specified types of cancer	Other & unspecified (O&U)	9064	Other specified types of cancer	Other & unspecified (O&U)
8041	Other specific carcinomas	Small cell carcinoma (SCLC)	8252	Other specific carcinomas	Adenocarcinoma (AC)	8770	Other specified types of cancer	Other & unspecified (O&U)	9065	Other specified types of cancer	Other & unspecified (O&U)
8042	Other specific carcinomas	Small cell carcinoma (SCLC)	8253	Other specific carcinomas	Adenocarcinoma (AC)	8772	Other specified types of cancer	Other & unspecified (O&U)	9070	Other specified types of cancer	Other & unspecified (O&U)
8043	Other specific carcinomas	Small cell carcinoma (SCLC)	8254	Other specific carcinomas	Adenocarcinoma (AC)	8800	Sarcomas and soft tissue tumours	Other & unspecified (O&U)	9071	Other specified types of cancer	Other & unspecified (O&U)
8044	Other specific carcinomas	Small cell carcinoma (SCLC)	8255	Other specific carcinomas	Adenocarcinoma (AC)	8801	Sarcomas and soft tissue tumours	Other & unspecified (O&U)	9080	Other specified types of cancer	Other & unspecified (O&U)
8045	Other specific carcinomas	Small cell carcinoma (SCLC)	8260	Adenocarcinomas	Adenocarcinoma (AC)	8802	Sarcomas and soft tissue tumours	Other & unspecified (O&U)	9084	Other specified types of cancer	Other & unspecified (O&U)
8046	Other specific carcinomas	Other & unspecified (O&U)	8263	Adenocarcinomas	Other & unspecified (O&U)	8803	Sarcomas and soft tissue tumours	Other & unspecified (O&U)	9085	Other specified types of cancer	Other & unspecified (O&U)
8050	Unspecified carcinomas (NOS)	Squamous cell carcinoma (SCC)	8290	Adenocarcinomas	Other & unspecified (O&U)	8804	Sarcomas and soft tissue tumours	Other & unspecified (O&U)	9100	Other specified types of cancer	Other & unspecified (O&U)
8051	Squamous carcinomas	Squamous cell carcinoma (SCC)	8310	Adenocarcinomas	Large cell carcinoma (LCC)	8805	Sarcomas and soft tissue tumours	Other & unspecified (O&U)	9101	Other specified types of cancer	Other & unspecified (O&U)
8052	Squamous carcinomas	Squamous cell carcinoma (SCC)	8320	Adenocarcinomas	Other & unspecified (O&U)	8806	Sarcomas and soft tissue tumours	Other & unspecified (O&U)	9120	Sarcomas and soft tissue tumours	Other & unspecified (O&U)
8070	Squamous carcinomas	Squamous cell carcinoma (SCC)	8323	Adenocarcinomas	Adenocarcinoma (AC)	8810	Sarcomas and soft tissue tumours	Other & unspecified (O&U)	9130	Sarcomas and soft tissue tumours	Other & unspecified (O&U)
8071	Squamous carcinomas	Squamous cell carcinoma (SCC)	8332	Adenocarcinomas	Other & unspecified (O&U)	8811	Sarcomas and soft tissue tumours	Other & unspecified (O&U)	9133	Sarcomas and soft tissue tumours	Other & unspecified (O&U)
8072	Squamous carcinomas	Squamous cell carcinoma (SCC)	8333	Adenocarcinomas	Other & unspecified (O&U)	8815	Sarcomas and soft tissue tumours	Other & unspecified (O&U)	9150	Sarcomas and soft tissue tumours	Other & unspecified (O&U)
8073	Squamous carcinomas	Squamous cell carcinoma (SCC)	8340	Other specific carcinomas	Other & unspecified (O&U)	8824	Sarcomas and soft tissue tumours	Other & unspecified (O&U)	9180	Sarcomas and soft tissue tumours	Unusual
8074	Squamous carcinomas	Squamous cell carcinoma (SCC)	8341	Other specific carcinomas	Other & unspecified (O&U)	8830	Sarcomas and soft tissue tumours	Other & unspecified (O&U)	9181	Sarcomas and soft tissue tumours	Other & unspecified (O&U)
8075	Squamous carcinomas	Squamous cell carcinoma (SCC)	8345	Other specific carcinomas	Other & unspecified (O&U)	8840	Sarcomas and soft tissue tumours	Other & unspecified (O&U)	9182	Sarcomas and soft tissue tumours	Other & unspecified (O&U)
8076	Squamous carcinomas	Squamous cell carcinoma (SCC)	8350	Adenocarcinomas	Other & unspecified (O&U)	8850	Sarcomas and soft tissue tumours	Other & unspecified (O&U)	9220	Sarcomas and soft tissue tumours	Unusual
8078	Squamous carcinomas	Squamous cell carcinoma (SCC)	8401	Adenocarcinomas	Other & unspecified (O&U)	8851	Sarcomas and soft tissue tumours	Other & unspecified (O&U)	9231	Sarcomas and soft tissue tumours	Other & unspecified (O&U)
8082	Squamous carcinomas	Other & unspecified (O&U)	8410	Adenocarcinomas	Other & unspecified (O&U)	8852	Sarcomas and soft tissue tumours	Other & unspecified (O&U)	9250	Sarcomas and soft tissue tumours	Other & unspecified (O&U)
8083	Squamous carcinomas	Squamous cell carcinoma (SCC)	8430	Adenocarcinomas	Other & unspecified (O&U)	8853	Sarcomas and soft tissue tumours	Other & unspecified (O&U)	9260	Other specified types of cancer	Unusual
8084	Squamous carcinomas	Squamous cell carcinoma (SCC)	8440	Adenocarcinomas	Other & unspecified (O&U)	8854	Sarcomas and soft tissue tumours	Other & unspecified (O&U)	9364	Other specified types of cancer	Other & unspecified (O&U)
8090	Basal cell carcinomas	Other & unspecified (O&U)	8441	Adenocarcinomas	Other & unspecified (O&U)	8855	Sarcomas and soft tissue tumours	Other & unspecified (O&U)	9365	Other specified types of cancer	Other & unspecified (O&U)
8094	Basal cell carcinomas	Other & unspecified (O&U)	8460	Adenocarcinomas	Other & unspecified (O&U)	8858	Sarcomas and soft tissue tumours	Other & unspecified (O&U)	9370	Sarcomas and soft tissue tumours	Other & unspecified (O&U)
8095	Basal cell carcinomas	Other & unspecified (O&U)	8470	Adenocarcinomas	Other & unspecified (O&U)	8890	Sarcomas and soft tissue tumours	Other & unspecified (O&U)	9473	Other specified types of cancer	Other & unspecified (O&U)
8120	Squamous carcinomas	Other & unspecified (O&U)	8471	Adenocarcinomas	Other & unspecified (O&U)	8891	Sarcomas and soft tissue tumours	Other & unspecified (O&U)	9500	Other specified types of cancer	Other & unspecified (O&U)
8123	Squamous carcinomas	Other & unspecified (O&U)	8480	Adenocarcinomas	Adenocarcinoma (AC)	8894	Sarcomas and soft tissue tumours	Other & unspecified (O&U)	9522	Other specified types of cancer	Other & unspecified (O&U)
8140	Adenocarcinomas	Adenocarcinoma (AC)	8481	Adenocarcinomas	Adenocarcinoma (AC)	8895	Sarcomas and soft tissue tumours	Other & unspecified (O&U)	9540	Sarcomas and soft tissue tumours	Other & unspecified (O&U)
8141	Adenocarcinomas	Other & unspecified (O&U)	8490	Adenocarcinomas	Adenocarcinoma (AC)	8896	Sarcomas and soft tissue tumours	Other & unspecified (O&U)	9560	Sarcomas and soft tissue tumours	Other & unspecified (O&U)
8143	Adenocarcinomas	Other & unspecified (O&U)	8500	Adenocarcinomas	Other & unspecified (O&U)	8900	Sarcomas and soft tissue tumours	Other & unspecified (O&U)	9561	Sarcomas and soft tissue tumours	Other & unspecified (O&U)

Notes: This classification is based on Fritz et al. 2013 (8) in combination with ICD-O-3 SEER Site/Histology Validation List 2015 to determine unusual codes for site Lung and Bronchus.

Section S6: Details on simulations to estimate the size of bias using standard SIR

We simulate various scenarios to estimate the size of bias introduced by using general population reference rates for calculating SIR of same-site SPC when IARC/IACR MP rules are applied. First, we assume that the baseline risk of LC survivors to develop an SPLC is the same as for the general population (real SIR = 1.0). We determined the proportions of histologically different LC groups p_{hist_j} in the analysis dataset for all index LC cases aged 30 to 99 years and excluded death certificate only (DCO) diagnoses. Then, we assumed that the SPLC would have the same histology group distribution as the first cancer. We expect the true SIR to be the fraction of observed and expected cases. In the case of the no risk difference between LC survivors and the general population SIR_{real}, the count of observed cases $count_i$ equals the number of expected cases (as the product of person-years at risk $pyears_i$ and general population reference rates IR_i) for each specific stratum i. We always stratified SIR in our analyses by age, sex, region, and period using stratum-specific reference rates for the general population.

$$SIR = \frac{O}{E} = \frac{\sum_{i=1}^{I} O_i}{\sum_{i=1}^{I} E_i} = \frac{\sum_{i=1}^{I} c\,ount_i}{\sum_{i=1}^{I} p\,yars_i * IR_i}$$

$$SIR_{real}(1.0) = \frac{\sum_{i=1}^{I} 1 * E_i}{\sum_{i=1}^{I} E_i}$$

Then, we take into account that there is a correction factor x_{hist} for combinations of LC and SPLC that are not possible in our observed cases according to IARC/IACR MP rules. Suppose we assume that the SPLC would have the same histology group distribution as for the first cancer, and any histology group A can only be followed by a histology group, not A. In that case, the correction factor is $1 - p_{hist_A}$. This gives for the simulated SIR under IARC/IACR MP rules:

$$SIR_{simIARC}(SIR_{real} = 1.0) = \frac{O}{E} = \frac{\sum_{j=1}^{J} \sum_{i=1}^{I} 1 * E_{ij} * x_{hist_j}}{\sum_{j=1}^{J} \sum_{i=1}^{I} E_{ij}}$$

Whereby

$$x_{hist_j} = 1 - p_{hist_j}$$

The factor x_{hist_j} is sex- and histology-specific, but the same for all age-groups and regions.

Generalized for any given SIR_{real}, the simulation would give

$$SIR_{simIARC} = \frac{\sum_{j=1}^{J} \sum_{i=1}^{I} S\,IR_{real} * pyears_{ij} * IR_{ij} * x_{hist_j}}{\sum_{j=1}^{J} \sum_{i=1}^{I} p\,years_{ij} * IR_{ij}}$$

Additionally to the scenario of no risk difference ($SIR_{real} = 1.0$), we also simulate a true doubling of SPLC risk for LC survivors ($SIR_{real} = 2.0$) and a risk increase comparable to data of U.S. lung cancer survivors for males ($SIR_{real} = 3.38$) and females ($SIR_{real} = 4.85$) published by Thakur et al.[6]

6. Thakur MK, Ruterbusch JJ, Schwartz AG, Gadgeel SM, Beebe-Dimmer JL, Wozniak AJ. Risk of Second Lung Cancer in Patients with Previously Treated Lung Cancer: Analysis of Surveillance, Epidemiology, and End Results (SEER) Data. Journal of Thoracic Oncology 2018; 13: 46–53.

Figure S7: Histological groups of LC and SPLC

Fig. S7: Histological groups of LC and SPLC

Groups of malignant neoplasms considered to be histologically 'different' for the purpose of defining multiple tumors (IARC/IACR definition, ICD-O-3 first revision)

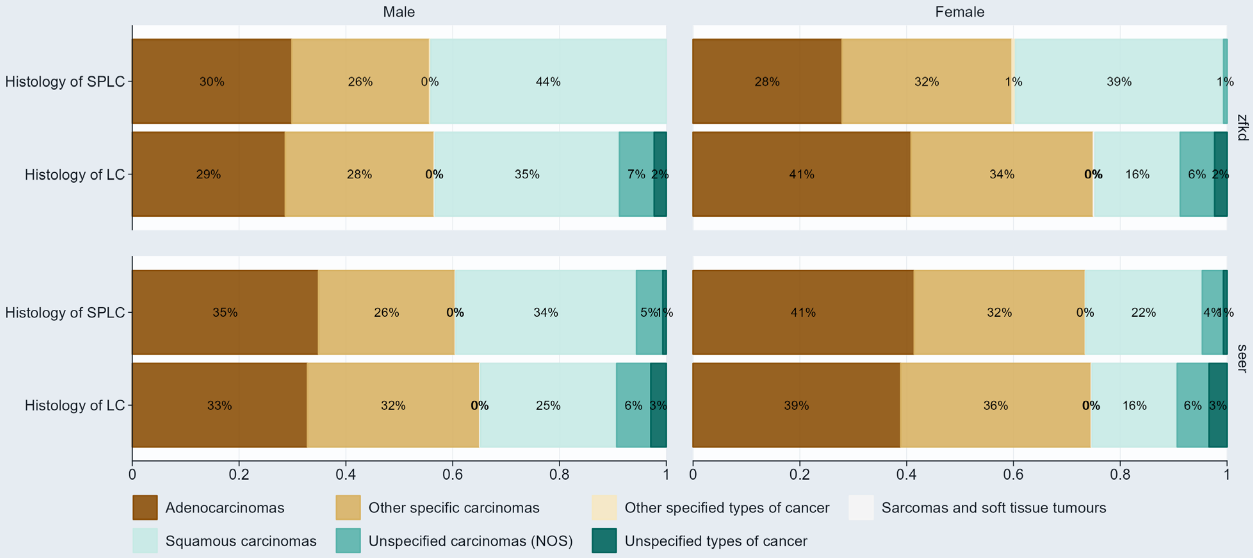

Table S8: Frequency of same-histology SPLC by region

Table S8: Frequency of same-histology SPLC by region			
**Regional Registry**	**Same group (%)**	**Different group (%)**	**Total (%)**
**Analysis Dataset – Germany (11 PBCR)**			
DEF Schleswig-Holstein	0	97 (100.0%)	97 (100.0%)
DE6 Hamburg	0	7 (100.0%)	7 (100.0%)
DE9 Lower Saxony	0	53 (100.0%)	53 (100.0%)
DE5 Bremen	0	18 (100.0%)	18 (100.0%)
DEA3 Muenster	0	77 (100.0%)	77 (100.0%)
DE2 Bavaria	0	87 (100.0%)	87 (100.0%)
DE4 Brandenburg	0	67 (100.0%)	67 (100.0%)
DED Saxony	0	61 (100.0%)	61 (100.0%)
DEG Thuringia	0	43 (100.0%)	43 (100.0%)
DE8 Mecklenburg-Western Pomerania	0	32 (100.0%)	32 (100.0%)
**Validation Dataset – United States (17 PBCR)**			
SEER Reg 01 - San Francisco-Oakland SMSA	107 (44.6%)	133 (55.4%)	240 (100.0%)
SEER Reg 02 - Connecticut	188 (40.9%)	272 (59.1%)	460 (100.0%)
SEER Reg 20 - Detroit (Metropolitan)	237 (46.4%)	274 (53.6%)	511 (100.0%)
SEER Reg 21 - Hawaii	45 (49.5%)	46 (50.5%)	91 (100.0%)
SEER Reg 22 - Iowa	134 (38.4%)	215 (61.6%)	349 (100.0%)
SEER Reg 23 - New Mexico	31 (47.7%)	34 (52.3%)	65 (100.0%)
SEER Reg 25 - Seattle (Puget Sound)	190 (44.3%)	239 (55.7%)	429 (100.0%)
SEER Reg 26 - Utah	26 (54.2%)	22 (45.8%)	48 (100.0%)
SEER Reg 27 - Atlanta (Metropolitan)	97 (44.5%)	121 (55.5%)	218 (100.0%)
SEER Reg 29 - Alaska Natives	3 (37.5%)	5 (62.5%)	8 (100.0%)
SEER Reg 31 - San Jose-Monterey	52 (50.0%)	52 (50.0%)	104 (100.0%)
SEER Reg 35 - Los Angeles	214 (46.0%)	251 (54.0%)	465 (100.0%)
SEER Reg 37 - Rural Georgia	3 (27.3%)	8 (72.7%)	11 (100.0%)
SEER Reg 41 - California excluding SF/SJM/LA	607 (47.0%)	684 (53.0%)	1291 (100.0%)
SEER Reg 42 - Kentucky	389 (42.5%)	527 (57.5%)	916 (100.0%)
SEER Reg 44 - New Jersey	388 (39.9%)	585 (60.1%)	973 (100.0%)
SEER Reg 47 - Georgia excluding Atlanta/Rural Georgia	287 (41.1%)	411 (58.9%)	698 (100.0%)

Groups of malignant neoplasms considered to be histologically ‘different’ according to IARC ICD-O-3, revision 1 (2013).

Figure S9: Relative risk for SPLC in lung cancer survivors stratified by follow-up time

Fig. S9: Relative risk for SPLC in lung cancer survivors stratified by follow-up time (n=399,394).

SIRs stratified by sex on the log-transformed y axis (for females in yellow and for males in blue) and stratified by follow-up time on the x axis. Top row shows values for unadjusted estimation of risk for SPLC after LC using general reference rates ($SIR1_{raw}$).
Bottom row shows SIR using subtype-specific reference rates excluding same-histology group ($SIR2_{sub}$).

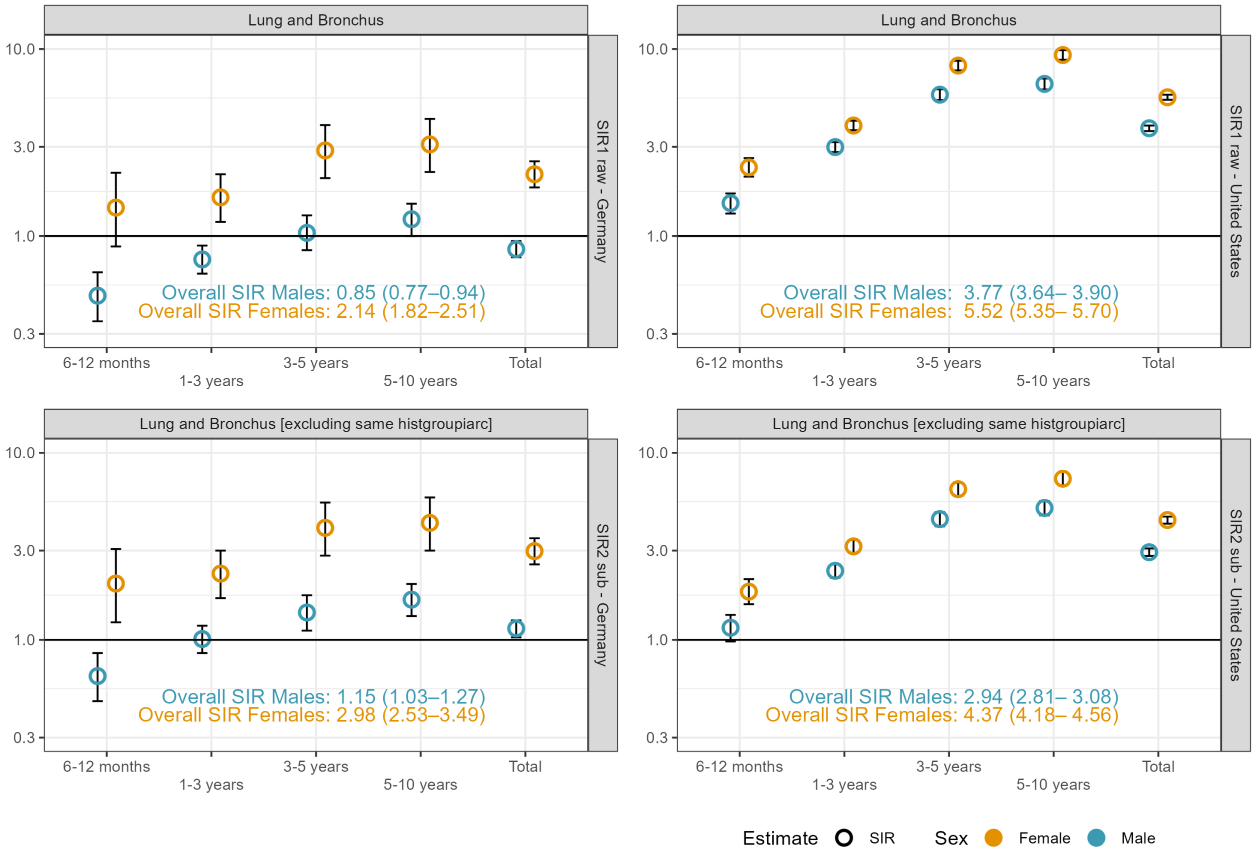

Notes: Numeric SIR values are given for total follow-up time (6 mo to 10+ years). SIR Standardized incidence ratio; length of error bar indicates 95% CI.

Table S10: Sensitivity analysis A – Risk of SPLC using unadjusted and histology-specific SIR method [restricted to six German PBCR with low DCO rate]

S10. Table: Sensitivity analysis A – Risk for SPLC using unadjusted and histology-specific SIR method [restricted to six German PBCR with low DCO rate]

Comparing results for Germany (IARC/IACR MP rules) and United States (Verification dataset - SEER MP rules)

	**Germany (6 of 11 regions)** (Sensitivity dataset - IARC/IACR MP rules)				**Difference to main analysis**		**Germany (all regions)** (Main analysis dataset - IARC/IACR MP rules)			**United States** (Validation dataset - SEER MP rules)		
	$SIR1_{raw}$	**$SIR2_{sub}$**	95% CI_{SIR2}	O_{SIR2}	Δ $SIR1_{raw}$	**Δ $SIR2_{sub}$**	$SIR1_{raw}$	**$SIR2_{sub}$**	95% CI_{SIR2}	$SIR1_{raw}$	**$SIR2_{sub}$**	95% CI_{SIR2}
**Females**												
Total - All lung cancers	1.64	**2.30**	1.50–3.37	26	−0.50	**−0.68**	2.14	**2.98**	2.53–3.49	5.52	**4.37**	4.18–4.56
Histology of LC												
Adenocarcinoma (AC)	1.05	**1.58**	0.68–3.11	8	−0.64	**−0.95**	1.69	**2.53**	1.91–3.28	6.08	**4.48**	4.20–4.76
Large cell carcinoma (LCC)	1.44	**1.69**	0.04–9.40	x	1.20	**1.40**	0.24	**0.29**	0.01–1.60	4.04	**3.95**	3.27–4.73
Other & unspecified (O&U)	1.18	**1.67**	0.34–4.88	x	−0.10	**−0.13**	1.28	**1.80**	0.98–3.01	4.04	**3.88**	3.49–4.30
Small cell carcinoma (SCLC)	2.92	**4.29**	1.58–9.35	6	0.49	**0.72**	2.43	**3.57**	2.26–5.35	4.26	**4.51**	3.79–5.32
Squamous cell carcinoma (SCC)	2.71	**3.25**	1.41–6.41	8	−1.64	**−1.92**	4.35	**5.17**	3.94–6.67	6.50	**4.66**	4.26–5.09
Age at diagnosis of LC												
30 - 49	5.92	**9.32**	1.13–33.68	x	1.72	**2.93**	4.20	**6.39**	2.57–13.17	37.95	**32.88**	26.97–39.70
50 - 59	3.28	**4.95**	2.14–9.75	8	−0.28	**−0.27**	3.56	**5.22**	3.79–7.00	14.29	**12.06**	10.85–13.37
60 - 69	1.28	**1.82**	0.73–3.75	7	−1.09	**−1.53**	2.37	**3.35**	2.59–4.26	7.23	**5.94**	5.54–6.36
70 - 79	1.50	**2.05**	0.94–3.89	9	0.16	**0.24**	1.34	**1.81**	1.24–2.55	3.90	**3.06**	2.83–3.30
80+	0.00	**0.00**	0.00–2.96	0	−0.82	**−1.06**	0.82	**1.06**	0.34–2.47	2.05	**1.55**	1.30–1.83
Year of diagnosis of LC												
2002 - 2005	1.00	**1.37**	0.28–4.01	x	−1.27	**−1.70**	2.27	**3.07**	2.31–3.99	5.71	**4.44**	4.15–4.74
2006 - 2009	1.76	**2.46**	1.31–4.21	13	−0.15	**−0.20**	1.91	**2.66**	1.99–3.48	5.61	**4.48**	4.18–4.80
2010 - 2013	1.82	**2.60**	1.25–4.78	10	−0.49	**−0.73**	2.31	**3.33**	2.44–4.44	5.00	**4.02**	3.63–4.44
**Males**												
Total - All lung cancers	0.73	**0.99**	0.78–1.23	80	−0.12	**−0.16**	0.85	**1.15**	1.03–1.27	3.77	**2.94**	2.81–3.08
Histology of LC												
Adenocarcinoma (AC)	0.77	**1.03**	0.68–1.50	27	−0.16	**−0.19**	0.93	**1.22**	1.02–1.45	4.19	**3.13**	2.91–3.37
Large cell carcinoma (LCC)	0.00	**0.00**	0.00–0.76	0	−0.04	**−0.04**	0.04	**0.04**	0.00–0.24	2.79	**2.92**	2.42–3.48
Other & unspecified (O&U)	0.53	**0.68**	0.27–1.41	7	−0.27	**−0.36**	0.80	**1.04**	0.74–1.41	2.68	**2.58**	2.29–2.89
Small cell carcinoma (SCLC)	0.96	**1.28**	0.61–2.35	10	−0.07	**−0.08**	1.03	**1.36**	0.99–1.81	3.09	**3.38**	2.80–4.04
Squamous cell carcinoma (SCC)	0.79	**1.13**	0.79–1.56	36	−0.10	**−0.12**	0.89	**1.25**	1.07–1.46	4.22	**2.86**	2.62–3.11
Age at diagnosis of LC												
30 - 49	1.57	**2.20**	0.06–12.27	x	−0.07	**−0.04**	1.64	**2.24**	0.73–5.22	26.40	**22.55**	17.33–28.86
50 - 59	1.40	**1.94**	1.00–3.39	12	−0.21	**−0.26**	1.61	**2.20**	1.70–2.81	10.10	**8.01**	7.14–8.95
60 - 69	0.84	**1.16**	0.80–1.61	34	−0.32	**−0.41**	1.16	**1.57**	1.37–1.80	4.79	**3.74**	3.46–4.03
70 - 79	0.63	**0.86**	0.59–1.21	32	0.10	**0.15**	0.53	**0.71**	0.58–0.86	2.73	**2.16**	1.99–2.35
80+	0.10	**0.13**	0.00–0.73	x	−0.10	**−0.12**	0.20	**0.25**	0.10–0.51	1.60	**1.26**	1.06–1.49
Year of diagnosis of LC												
2002 - 2005	0.50	**0.68**	0.36–1.16	13	−0.29	**−0.37**	0.79	**1.05**	0.89–1.22	3.71	**2.92**	2.72–3.13
2006 - 2009	0.87	**1.17**	0.86–1.56	47	−0.07	**−0.09**	0.94	**1.26**	1.07–1.48	4.07	**3.15**	2.92–3.40
2010 - 2013	0.67	**0.92**	0.56–1.42	20	−0.17	**−0.23**	0.84	**1.15**	0.90–1.44	3.30	**2.59**	2.30–2.91

Notes: The six included registries are Brandenburg 2007 to 2014, Bremen 2004 to 2014, Hamburg 2008 to 2014, Mecklenburg-Western Pomerania 2003 to 2011, Saarland 2002 to 2011 and Saxony 2005 to 2014. O_{SIR1} number of cases observed in the data for $SIR1_{raw}$; O_{SIR2} number of cases observed in the data for $SIR2_{sub}$, ZfKD data O_{SIR1} = O_{SIR2}; SEER Surveillance, Epidemiology, and End Results Program; SIR standardized incidence ratio; $SIR1_{raw}$ unadjusted SIR using age-, sex-, region-, period-specific reference rates; $SIR2_{sub}$ histological subtype-specific SIR using age-, sex-, region-, period- and histology-specific reference rates and excluding same-histology group SPLC from observed and expected; $SIR3_{IARC}$ unadjusted SIR but only counting international primaries (IARC/IACR MP rules), for ZfKD data $SIR1_{raw}$ = $SIR3_{IARC}$; $SIR4_{subIARC}$ histological subtype-specific SIR but only counting international primaries (IARC/IACR MP rules), for ZfKD data $SIR2_{sub}$ = $SIR4_{subIARC}$; SPLC second primary lung cancer; x censored counts of observed smaller than 5 for data privacy reasons; ZfKD German Centre for Cancer Registry Data

Table S11: Sensitivity analysis B – Risk of SPLC using unadjusted and histology-specific SIR method [SEER restricted to White population]

S11. Table: Sensitivity analysis B – Risk for SPLC using unadjusted and histology-specific SIR method
[SEER restricted to White population]

Comparing results for Germany (IARC/IACR MP rules) and United States (Sensitivity dataset - SEER MP rules)

	Germany (Analysis dataset - IARC/IACR MP rules)				United States (White) (Sensitivity dataset - SEER MP rules)							Difference to main analysis (US White - All races)		United States (All races) (Validation dataset - SEER MP rules)		
	$SIR1_{raw}$	**$SIR2_{sub}$**	95% CI_{SIR2}	O_{SIR2}	$SIR1_{raw}$	**$SIR2_{sub}$**	95% CI_{SIR2}	$SIR3_{IARC}$	$SIR4_{subIARC}$	O_{SIR1}	O_{SIR2}	Δ $SIR1_{raw}$	**Δ $SIR2_{sub}$**	$SIR1_{raw}$	**$SIR2_{sub}$**	95% CI_{SIR2}
**Females**																
Total - All lung cancers	2.14	**2.98**	2.53–3.49	154	5.46	**4.30**	4.11–4.50	2.48	3.50	3,328	1,858	−0.06	**−0.07**	5.52	**4.37**	4.18–4.56
Histology of LC																
Adenocarcinoma (AC)	1.69	**2.53**	1.91–3.28	57	6.04	**4.40**	4.11–4.70	2.59	3.89	1,800	872	−0.04	**−0.08**	6.08	**4.48**	4.20–4.76
Large cell carcinoma (LCC)	0.24	**0.29**	0.01–1.60	x	4.24	**4.23**	3.48–5.09	0.33	0.37	128	112	0.20	**0.28**	4.04	**3.95**	3.27–4.73
Other & unspecified (O&U)	1.28	**1.80**	0.98–3.01	14	3.94	**3.76**	3.35–4.20	2.02	2.94	481	315	−0.10	**−0.12**	4.04	**3.88**	3.49–4.30
Small cell carcinoma (SCLC)	2.43	**3.57**	2.26–5.35	23	4.24	**4.53**	3.77–5.39	2.53	3.99	187	127	−0.02	**0.02**	4.26	**4.51**	3.79–5.32
Squamous cell carcinoma (SCC)	4.35	**5.17**	3.94–6.67	59	6.38	**4.54**	4.13–4.99	3.25	3.92	732	432	−0.12	**−0.12**	6.50	**4.66**	4.26–5.09
Age at diagnosis of LC																
30 - 49	4.20	**6.39**	2.57–13.17	7	38.65	**32.92**	26.29–40.70	16.32	25.68	157	85	0.70	**0.04**	37.95	**32.88**	26.97–39.70
50 - 59	3.56	**5.22**	3.79–7.00	44	14.61	**11.97**	10.65–13.40	6.65	10.12	559	301	0.32	**−0.09**	14.29	**12.06**	10.85–13.37
60 - 69	2.37	**3.35**	2.59–4.26	66	7.25	**5.94**	5.52–6.39	3.34	4.85	1,291	728	0.02	**0.00**	7.23	**5.94**	5.54–6.36
70 - 79	1.34	**1.81**	1.24–2.55	32	3.90	**3.06**	2.82–3.31	1.74	2.44	1,102	617	0.00	**0.00**	3.90	**3.06**	2.83–3.30
80+	0.82	**1.06**	0.34–2.47	5	2.07	**1.60**	1.33–1.90	0.97	1.29	219	127	0.02	**0.05**	2.05	**1.55**	1.30–1.83
Year of diagnosis of LC																
2002 - 2005	2.27	**3.07**	2.31–3.99	55	5.62	**4.39**	4.10–4.71	2.59	3.61	1,436	804	−0.09	**−0.05**	5.71	**4.44**	4.15–4.74
2006 - 2009	1.91	**2.66**	1.99–3.48	53	5.58	**4.42**	4.10–4.75	2.56	3.62	1,289	722	−0.03	**−0.06**	5.61	**4.48**	4.18–4.80
2010 - 2013	2.31	**3.33**	2.44–4.44	46	4.92	**3.89**	3.48–4.33	2.12	3.04	603	332	−0.08	**−0.13**	5.00	**4.02**	3.63–4.44
**Males**																
Total - All lung cancers	0.85	**1.15**	1.03–1.27	388	3.79	**2.91**	2.77–3.07	1.71	2.33	2,651	1,498	0.02	**−0.03**	3.77	**2.94**	2.81–3.08
Histology of LC																
Adenocarcinoma (AC)	0.93	**1.22**	1.02–1.45	132	4.21	**3.08**	2.84–3.34	1.97	2.77	1,136	591	0.02	**−0.05**	4.19	**3.13**	2.91–3.37
Large cell carcinoma (LCC)	0.04	**0.04**	0.00–0.24	x	2.68	**2.78**	2.26–3.39	0.27	0.31	108	98	−0.11	**−0.14**	2.79	**2.92**	2.42–3.48
Other & unspecified (O&U)	0.80	**1.04**	0.74–1.41	40	2.68	**2.55**	2.24–2.89	1.35	1.87	351	241	0.00	**−0.03**	2.68	**2.58**	2.29–2.89
Small cell carcinoma (SCLC)	1.03	**1.36**	0.99–1.81	46	3.03	**3.29**	2.68–4.00	2.05	3.02	135	100	−0.06	**−0.09**	3.09	**3.38**	2.80–4.04
Squamous cell carcinoma (SCC)	0.89	**1.25**	1.07–1.46	169	4.29	**2.89**	2.63–3.16	1.80	2.38	921	468	0.07	**0.03**	4.22	**2.86**	2.62–3.11
Age at diagnosis of LC																
30 - 49	1.64	**2.24**	0.73–5.22	5	29.60	**24.40**	18.17–32.08	14.74	20.96	88	51	3.20	**1.85**	26.40	**22.55**	17.33–28.86
50 - 59	1.61	**2.20**	1.70–2.81	65	10.80	**8.38**	7.36–9.50	4.60	6.44	441	244	0.70	**0.37**	10.10	**8.01**	7.14–8.95
60 - 69	1.16	**1.57**	1.37–1.80	207	4.90	**3.77**	3.47–4.09	2.17	3.00	1,023	568	0.11	**0.03**	4.79	**3.74**	3.46–4.03
70 - 79	0.53	**0.71**	0.58–0.86	104	2.76	**2.16**	1.98–2.35	1.30	1.78	903	519	0.03	**0.00**	2.73	**2.16**	1.99–2.35
80+	0.20	**0.25**	0.10–0.51	7	1.63	**1.26**	1.04–1.52	0.70	0.92	196	116	0.03	**0.00**	1.60	**1.26**	1.06–1.49
Year of diagnosis of LC																
2002 - 2005	0.79	**1.05**	0.89–1.22	156	3.73	**2.87**	2.66–3.10	1.70	2.29	1,160	662	0.02	**−0.05**	3.71	**2.92**	2.72–3.13
2006 - 2009	0.94	**1.26**	1.07–1.48	157	4.05	**3.09**	2.84–3.35	1.79	2.45	1,052	588	−0.02	**−0.06**	4.07	**3.15**	2.92–3.40
2010 - 2013	0.84	**1.15**	0.90–1.44	75	3.40	**2.67**	2.35–3.02	1.57	2.19	439	248	0.10	**0.08**	3.30	**2.59**	2.30–2.91

Notes: This sensitivity analysis replicates Table 3, with SEER data restricted to White population only.
O_{SIR1} number of cases observed in the data for $SIR1_{raw}$; O_{SIR2} number of cases observed in the data for $SIR2_{sub}$, ZfKD data O_{SIR1} = O_{SIR2}; SEER Surveillance, Epidemiology, and End Results Program; SIR standardized incidence ratio; $SIR1_{raw}$ unadjusted SIR using age-, sex-, region-, period-specific reference rates; $SIR2_{sub}$ histological subtype-specific SIR using age-, sex-, region-, period- and histology-specific reference rates and excluding same-histology group SPLC from observed and expected; $SIR3_{IARC}$ unadjusted SIR but only counting international primaries (IARC/IACR MP rules), for ZfKD data $SIR1_{raw}$ = $SIR3_{IARC}$; $SIR4_{subIARC}$ histological subtype-specific SIR but only counting international primaries (IARC/IACR MP rules), for ZfKD data $SIR2_{sub}$ = $SIR4_{subIARC}$; SPLC second primary lung cancer; x censored counts of observed smaller than 5 for data privacy reasons; ZfKD German Centre for Cancer Registry Data
